# Structure and mechanism of the mitochondrial calcium transporter NCLX

**DOI:** 10.1038/s41586-025-09491-0

**Published:** 2025-09-10

**Authors:** Minrui Fan, Chen-Wei Tsai, Jinru Zhang, Jianxiu Zhang, Aswini R. Krishnan, Tsung-Yun Liu, Yu-Lun Huang, Deniz Aydin, Siyuan Du, Briana L. Sobecks, Madison X. Rodriguez, Andrew H. Reiter, Carolyn R. Bertozzi, Ron O. Dror, Ming-Feng Tsai, Liang Feng

**Affiliations:** 1 Department of Molecular and Cellular Physiology, Stanford University School of Medicine, Stanford, CA USA; 2 Department of Physiology and Biophysics, University of Colorado Anschutz Medical Campus, Aurora, CO USA; 3 Department of Molecular Physiology and Biological Physics, University of Virginia, Charlottesville, VA USA; 4 Department of Computer Science, Stanford University, Stanford, CA USA; 5 Department of Structural Biology, Stanford University School of Medicine, Stanford, CA USA; 6 Institute for Computational and Mathematical Engineering, Stanford University, Stanford, CA USA; 7 Department of Chemistry, Stanford University, Stanford, CA USA; 8 Department of Chemical Engineering, Stanford University, Stanford, CA USA; 9 Department of Biology, Stanford University, Stanford, CA USA; 10 Stanford Sarafan ChEM-H, Stanford University, Stanford, CA USA

**Keywords:** Permeation and transport, Cryoelectron microscopy, Calcium and vitamin D, Transporters

## Abstract

As a key mitochondrial Ca^2+^ transporter, NCLX regulates intracellular Ca^2+^ signalling and vital mitochondrial processes^1–3^. The importance of NCLX in cardiac and nervous-system physiology is reflected by acute heart failure and neurodegenerative disorders caused by its malfunction^4–9^. Despite substantial advances in the field, the transport mechanisms of NCLX remain unclear. Here we report the cryo-electron microscopy structures of NCLX, revealing its architecture, assembly, major conformational states and a previously undescribed mechanism for alternating access. Functional analyses further reveal an unexpected transport function of NCLX as a H^+^/Ca^2+^ exchanger, rather than as a Na^+^/Ca^2+^ exchanger as widely believed^1^. These findings provide critical insights into mitochondrial Ca^2+^ homeostasis and signalling, offering clues for developing therapies to treat diseases related to abnormal mitochondrial Ca^2+^.

## Abstract (web summary)

Cryogenic electron microscopy structures and functional analyses reveal that NCLX functions as a H^+^/Ca^2+^ rather than a Na^+^/Ca^2+^ exchanger, and uncover its transport mechanism with implications for therapies treating cardiac and neurodegenerative disorders related to abnormal mitochondrial Ca^2+^.

## Main

Mitochondrial Ca^2+^ transport—which is critical for tuning cell bioenergetics, shaping cytosolic Ca^2+^ transients and regulating cell death pathways^10,11^—is mediated by (1) the mitochondrial Ca^2+^ uniporter (referred to hereafter as uniporter), a highly selective Ca^2+^ channel that delivers cytoplasmic Ca^2+^ into the mitochondrial matrix^12–14^, and (2) Ca^2+^ transporters that catalyse H^+^- or Na^+^-dependent Ca^2+^ efflux^15–17^. Although substantial progress has been made in mechanistic studies on the uniporter^2,11,18^, our understanding of the mechanisms and molecular identities of Ca^2+^-efflux transporters remains limited.

Mitochondrial Ca^2+^ efflux is essential for preventing mitochondrial Ca^2+^ overload, a detrimental condition that leads to cell death, and for protecting against pathological conditions such as heart failure, neuromuscular diseases and neurodegeneration^2^. The prevailing view is that NCLX—a member of the Ca^2+^/cation antiporter (CaCA) superfamily—mediates mitochondrial Na^+^/Ca^2+^ exchange (mito-NCX)^1–3^, extruding matrix Ca^2+^ into the cytoplasm^17^. The activity of NCLX is critical for normal physiology in numerous cell types, including cardiomyocytes^4^, neurons and astrocytes^19–21^, brown adipose tissue^22^, pancreatic β-cells^23^ and B lymphocytes^24^. Dysfunction of NCLX has been linked to heart failure^4,5^, neurodegenerative disorders^6–9^ and tumour progression^25,26^. Thus, elucidating NCLX’s structural and functional mechanisms will fundamentally improve our knowledge of physiology and disease.

There are currently two major knowledge gaps in NCLX mechanisms. First, the structural basis for its substrate recognition and transport remains unknown. Ca^2+^/cation antiporter proteins fall into five distinct families: Na^+^/Ca^2+^ exchangers (NCX), K^+^-dependent Na^+^/Ca^2+^ exchangers (NCKX), H^+^/Ca^2+^ exchangers (CAX), prokaryotic-specific YRBG and cation/Ca^2+^ exchangers (CCX), the family to which NCLX belongs^27^. Structures of NCX-family (archaeal NCX from *Methanococcus jannaschii*, *Mj*NCX, and recent human NCX1) and CAX-family (yeast Vcx1, archaeal CAX from *Archaeoglobus fulgidus*, *Af*CAX, and bacterial YfkE)^28–32^ transporters have provided important mechanistic insights. Nonetheless, the utility of these structures for understanding NCLX is limited due to their low sequence identity with NCLX and the presence of substantial structural elements unique to NCLX.

Moreover, the inward- and outward-facing conformations of CaCA proteins were captured from distant homologues^28–32^. The structural divergence associated with sequence and function divergence, combined with challenges in precisely aligning structural elements, hampers efforts to pinpoint exact conformational changes underlying the alternating-access mechanism. Although recent molecular dynamics simulations of *Mj*NCX have provided insights into its conformational cycle^33^, experimental evidence for such conformational changes remains unavailable. In this regard, obtaining both inward- and outward-facing structures of the same transporter could shed light on the molecular basis of ion exchange and translocation in NCLX and, more broadly, in CaCA proteins.

The second knowledge gap concerns NCLX function. Although proposed^1^ to mediate mito-NCX, NCLX, puzzlingly, lacks multiple Na^+^-coordinating residues conserved in NCX proteins^28,34^. Moreover, although *NCLX* knockout (KO) has been achieved in mouse models and cell lines, there has been no unambiguous demonstration that *NCLX* KO abolishes mito-NCX. Various studies using intact cells have assessed the impact of *NCLX* KO on mitochondrial Ca^2+^ efflux, which is mediated not only by mito-NCX but also by other Ca^2+^ transport mechanisms; however, the results have been conflicting, with effects ranging from profound to marginal^4,6,19,22,25,35,36^. There is therefore clearly a need to define NCLX’s function using more quantitative approaches.

Here we report the cryogenic electron microscopy (cryo-EM) structures of rat NCLX in cytosol-facing (intermembrane space) and matrix-facing conformations, with and without Ca^2+^. Together with functional investigations using quantitative flux assays in heterologous systems, our work provides a framework for understanding NCLX’s architecture, ion selectivity, transport mechanism and physiological functions.

## Overall structure

**Fig. 1:**
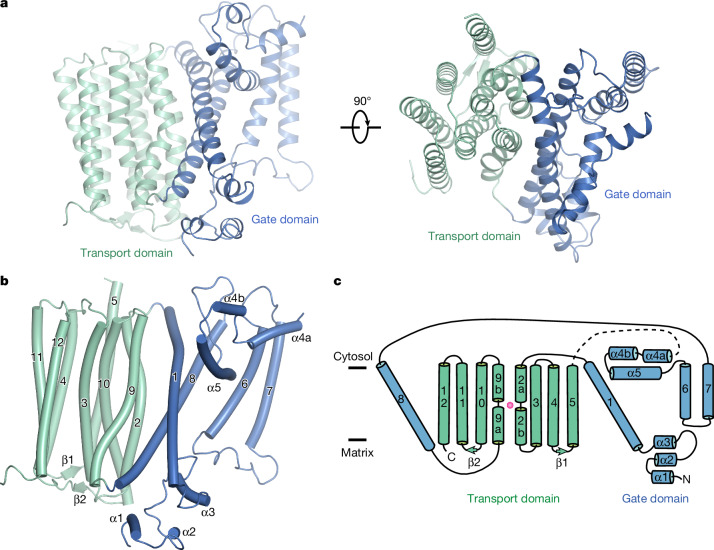
Structure of an NCLX protomer. **a**, Structure of an NCLX protomer. The ribbon representations of the NCLX protomer (in a matrix open conformation) are viewed from membrane and intermembrane space, respectively. **b**, The overall architecture of an NCLX protomer. **c**, The topology of NCLX.

**Extended Data Fig. 1:**
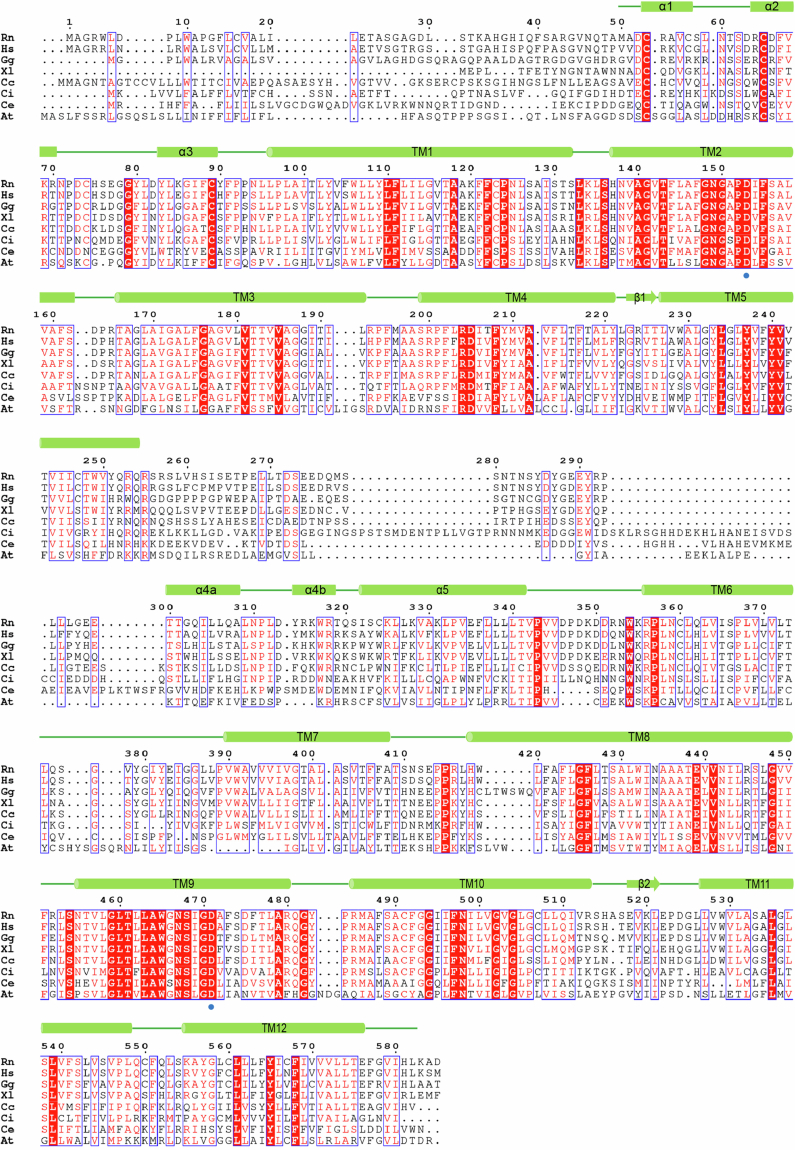
Sequence alignment of NCLX. The sequences of NCLX from *Rattus norvegicus* (Rn; NCBI-ProteinID: NP_001017488), *Homo sapiens* (Hs; ID: NP_079235), *Gallus gallus* (Gg; ID: XP_004934554), *Xenopus laevis* (Xl; ID: NP_001128697), *Carcharodon carcharias* (Cc; ID: XP_041058292), *Ciona intestinalis* (Ci; ID: XP_002130159), *Caenorhabditis elegans* (Ce; ID: NP_499146), *Arabidopsis thaliana* (At; ID: NP_197288) are aligned. For each organism, the protein with the highest sequence similarity to rat NCLX (identified as the top BLASTP hit) was selected for sequence alignment. These homologues all exhibit similarity across the full length of the protein. Subcellular localization of these homologues remains to be determined. Some organisms, such as *C. elegans*, possess multiple potential NCLX homologues, including ncx-9, which has been suggested to localize to mitochondria^90^. The two Ca^2+^-coordinating aspartate residues, D153 and D471, are indicated with blue circles.

**Extended Data Fig. 2:**
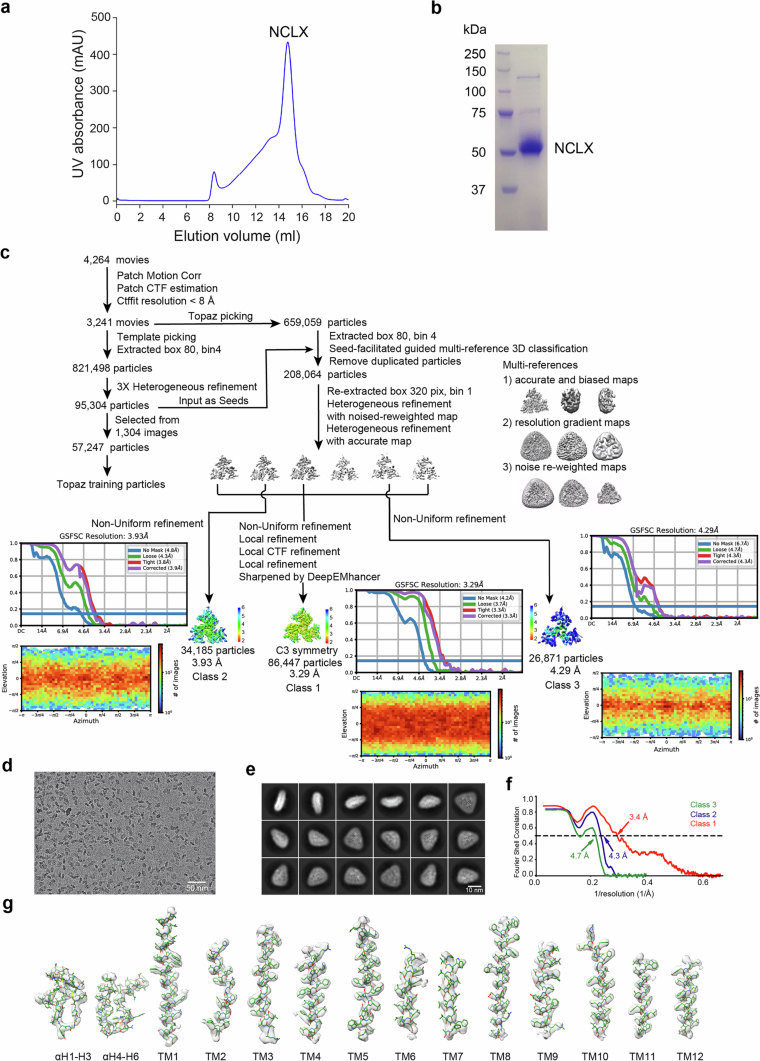
Purification and biochemical characterization of rat NCLX and cryo-EM data processing of NCLX without calcium (pH 7.4). **a**, Gel filtration profile of NCLX. **b**, SDS-PAGE gel of the purified NCLX (representative of six independent experiments with similar results). The gel source data are included in Supplementary Fig. 1. **c**, A flowchart of NCLX without calcium data processing. Conformational states of protomers: Class 1, three in matrix-facing conformation; Class 2, two in matrix-facing and one in cytosol-facing conformation; Class 3, one in matrix-facing and two in cytosol-facing conformation. Final map gold-standard FSC curves and angular particle distribution are shown next to the local resolution map for each class. **d**, A representative cryo-EM image (from 4,264 micrographs with similar results). **e**, Typical 2D class averages. **f**, Map vs. model FSC. **g**, Cryo-EM density corresponding to model.

**Extended Data Fig. 3:**
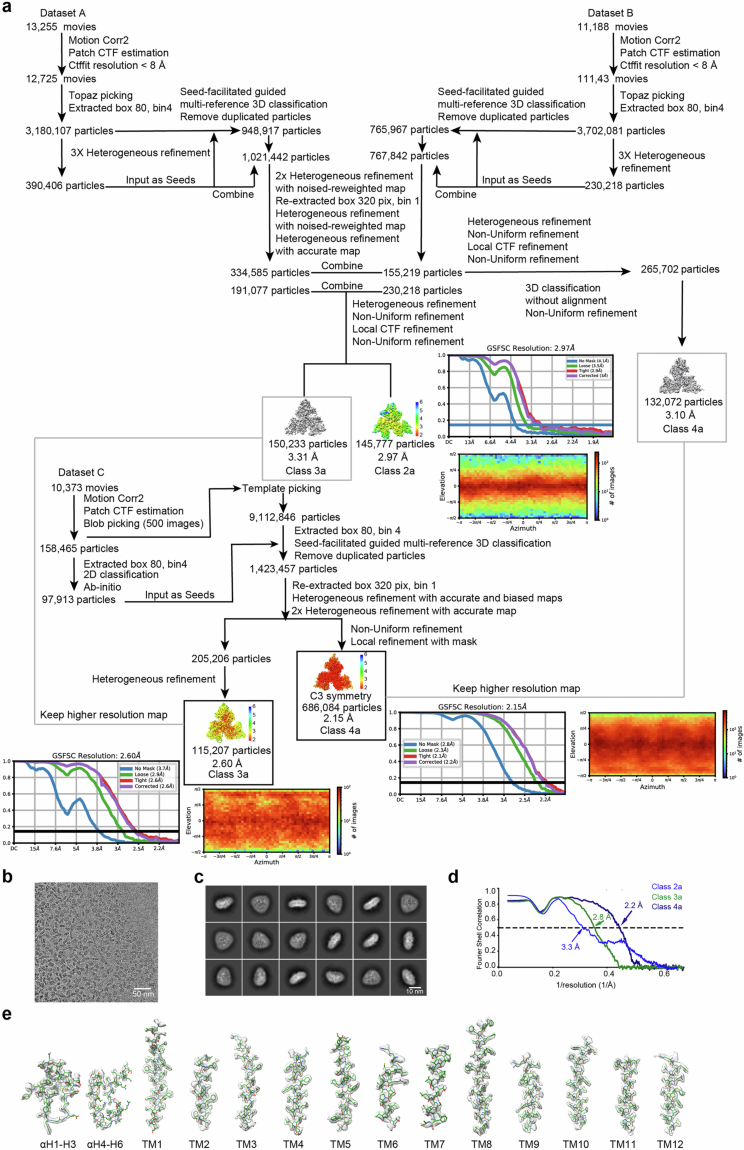
Cryo-EM data processing of NCLX with calcium. **a**, A flowchart of NCLX with calcium data processing. Conformational states of protomers: Class 2a, two in matrix-facing and one in cytosol-facing conformation; Class 3a, one in matrix-facing and two in cytosol-facing conformation; Class 4a, three in cytosol-facing conformation. Final map gold-standard FSC curves and angular particle distribution are shown next to the local resolution map for each class. **b**, A representative cryo-EM image (from 24,443 micrographs with similar results). **c**, Typical 2D class averages. **d**, Map vs. model FSC. **e**, Cryo-EM density corresponding to model.

**Extended Data Fig. 4:**
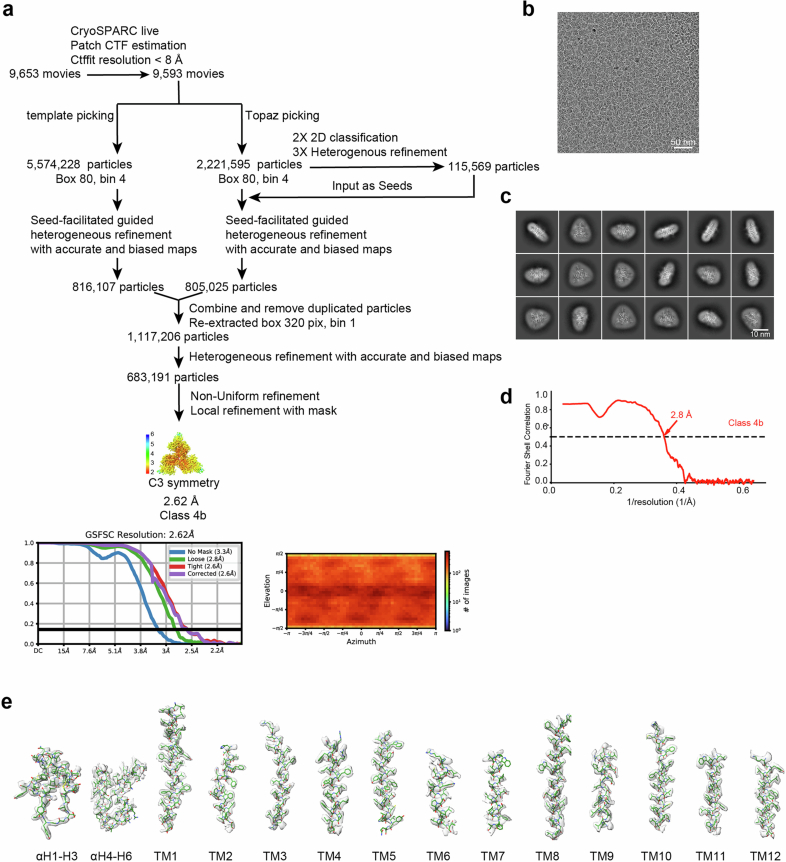
Cryo-EM data processing of NCLX without calcium at low pH. **a**, A flowchart of NCLX without calcium at low pH data processing. Conformational states of protomers: Class 4b, three in cytosol-facing conformation. Final map gold-standard FSC curves and angular particle distribution are shown next to the local resolution map. **b**, A representative cryo-EM image (from 9,653 micrographs with similar results). **c**, Typical 2D class averages. **d**, Map vs. model FSC. **e**, Cryo-EM density corresponding to model.

We selected rat NCLX (83% sequence identity with human NCLX; Extended Data Fig. 1) for cryo-EM due to its favourable biochemical behaviour (Extended Data Fig. 2a,b). Cryo-EM studies on NCLX under Ca^2+^-bound (2 mM Ca^2+^, pH 7.4) or Ca^2+^-free (5 mM ethylene glycol tetraacetic acid (EGTA), without adding Ca^2+^, pH 5.5 and 7.4) conditions produced maps with resolutions of up to 2.15 Å (Extended Data Figs. 2–4), enabling unambiguous modelling of NCLX (Fig. 1 and Extended Data Figs. 2–4).

**Fig. 2:**
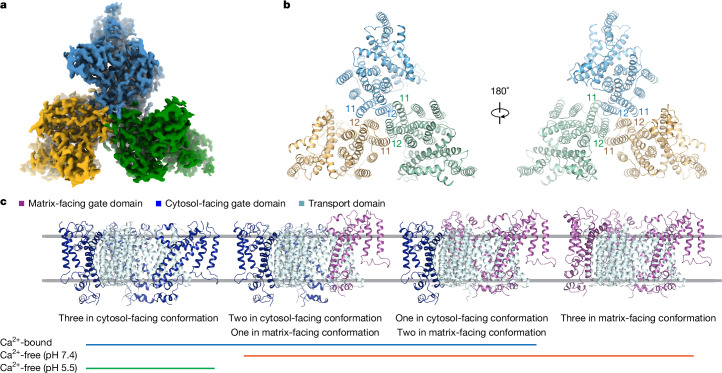
Assembly of NCLX. **a**, The density map of an NCLX trimer. The density map (Ca^2+^ bound, cytosol-facing conformation) is coloured by subunit. **b**, The structure of an NCLX trimer. The Ca^2+^ bound, cytosol-facing conformation of NCLX is shown as a ribbon from the cytosolic and matrix sides, respectively, with TM11 and TM12 labelled at the trimer interfaces. **c**, The conformational state assembly of NCLX trimers. The transport domain is coloured cyan, whereas the gate domain of each protomer is coloured according to the conformational state. The conditions leading to the 3D reconstructions with specific conformational state assembly are indicated by the coloured lines below.

Two-dimensional classifications showed that NCLX adopts a trimeric assembly in both Ca^2+^-bound and -free conditions (Extended Data Figs. 2–4), consistent with its relatively early elution volume on size-exclusion chromatography (Extended Data Fig. 2a,b). All three protomers are in the same orientation and are at the same plane level, compatible with a trimeric assembly in the membrane (Fig. 2a,b). Moreover, the same trimer assembly is maintained regardless of the protomer conformational states (Fig. 2c). The trimer interface is formed through interactions between transmembrane (TM) helices 11 and 12 of each subunit (Fig. 2b), with prominent crevices between protomers filled with non-protein densities, presumably lipids or detergent molecules.

**Extended Data Fig. 5:**
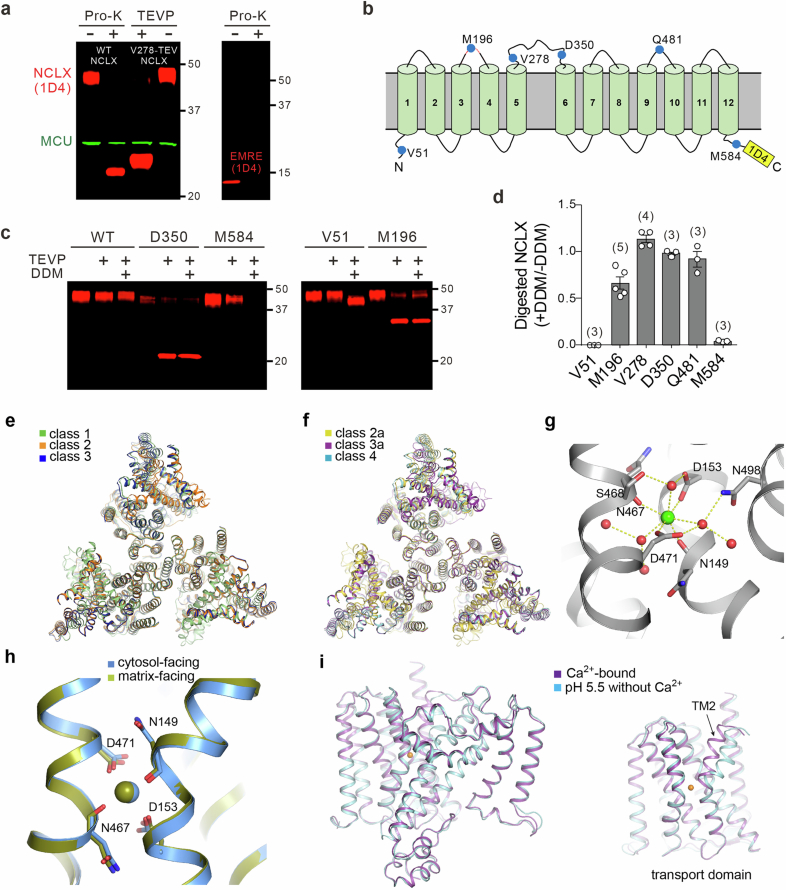
Determination of NCLX orientation and the comparison of NCLX assembly and the Ca^2+^-binding site in different conformational states. **a**, Protease digestion of NCLX. After isolating mitoplasts from HEK cells expressing C-terminally 1D4-tagged human NCLX, adding proteinase K (Pro-K) causes a downward shift of the NCLX band, as detected by an anti-1D4 antibody (image on the left). This suggests that (1) the C-terminal 1D4 tag dwells within the matrix and is therefore protected from Pro-K digestion, and (2) Pro-K cuts an NCLX area in the intermembrane space to cause the observed band shift. As the loop between TM5 and TM6 is large and unstructured, we hypothesized that this loop is digested by Pro-K. Accordingly, we introduced a TEV protease (TEVP) site after V278 in the TM5-6 loop and digested this construct in mitoplasts using TEVP. This manoeuvre produces a band at a similar location as the band generated by Pro-K digestion of WT NCLX, suggesting that Pro-K and TEVP both can cut the TM5-6 loop and that this loop is in the intermembrane space. Two subunits in the mitochondrial Ca^2+^ uniporter complex, MCU and EMRE, were used as controls. The green bands represent native MCU proteins. As most of MCU’s protein mass is in the matrix, it is not affected by Pro-K or TEVP. The EMRE protein has its C-terminal end exposed to the intermembrane space. Therefore, Pro-K was able to digest a 1D4 tag attached to EMRE’s C-terminus, as reflected by the disappearance of the Western blot signal created by the anti-1D4 antibody (image on the right). The experiment was performed with four independent biological replicates, all yielding similar results. Molecular weight marker unit: kDa. For gel source data, see Supplementary Fig. 1. **b**, A schematic of NCLX orientation and transmembrane topology. Key residues in panels **a** and **c** are highlighted in blue. The orange dotted lines surrounding M196 indicate linkers that were engineered to make M196 more exposed. **c**, TEVP digestion of NCLX. TEVP sites were introduced into NCLX in positions after the indicated residues. Those sites, whose digestion by TEVP is unaffected by DDM (e.g., D350 and M196), are located in the intermembrane space. By contrast, those sites, which require DDM to be fully digested by TEVP (e.g., M584 and V51), are inside the matrix. In the presence of DDM, cleavage of the TEVP site after M584 causes the disappearance of the band (lane 9 from the left). This is because the fragment that contains the 1D4 tag is too small (~10 amino acids) and would migrate out of the gel. Digestion of the TEVP site after V51 causes a band shift (lane 12 from the left). This is because the digested fragment that contains NCLX residue 52–584 and the C-terminal 1D4 tag is smaller than the undigested NCLX (lanes 10 and 11). For gel source data, see Supplementary Fig. 1. **d**, A summary of TEVP digestion results. The Western signal ratios of digested NCLX with or without DDM in panel **c** are presented. A ratio close to 1 indicates that DDM does not have effects on proteolysis, while a ratio close to 0 indicates that the TEV site is in the matrix, protected by the inner mitochondrial membrane. Data are shown as means ± s.e.m. Numbers in parentheses indicate the number of independent biological replicates. **e**, The superposition of three classes of Ca^2+^-free NCLX. The transport domains that mediate the oligomerization superimpose well. **f**, The superposition of three classes of NCLX with Ca^2+^. The transport domains that mediate the oligomerization superimpose well. **g**, The coordination environment around the Ca^2+^ binding site (cytosol-facing conformation). Ca^2+^ is depicted as a green sphere, and the water molecules are shown as red spheres. **h**, Superposition of Ca^2+^-coordinating residues in cytosol- and matrix- facing conformations. **i**, Superposition of cytosol-facing NCLX with Ca^2+^ bound or at low pH without Ca^2+^ (comparison of the protomer on the left and the transport domain on the right). Source Data

The orientation of NCLX in the inner mitochondrial membrane (IMM) is currently unknown. We performed protease digestion on human NCLX in HEK293 cell mitoplasts (submitochondrial vesicles with ruptured outer membranes) to investigate this. Two key observations support the conclusion that NCLX’s N and C termini—located on the same side of the protein—reside in the matrix. First, a C-terminal 1D4 tag is protected from proteinase K digestion (Extended Data Fig. 5a). Second, engineered tobacco etch virus (TEV) protease sites on the same side as the N and C termini are protected from TEV protease, whereas those on the opposite side are susceptible (Extended Data Fig. 5a–d). As a control, solubilizing the IMM exposes all previously inaccessible sites to digestion (Extended Data Fig. 5c). Together, these results define the side of NCLX containing the N and C termini as matrix-facing and the opposite side as cytosol-facing.

Each NCLX protomer contains twelve transmembrane helices and two sets of helical hairpins/bundles roughly parallel to the membrane (Fig. 1). Two transmembrane helices (TM6 and TM7) and five membrane-parallel helices (α1–5) are unique to NCLX, giving rise to a unique overall architecture. The structure comprises a transport domain, which mediates NCLX trimer formation at the centre, and a gate domain, located on the periphery without contacting other protomers. The transport domain includes an inner, intertwined four-helix bundle (TM2, TM3, TM9 and TM10), which contains the signature α-repeats critical for Ca^2+^ recognition in CaCA transporters, and a peripheral layer of TM4, TM5, TM11 and TM12. This domain shares a similar structural organization with corresponding regions^28–32^ in prokaryotic and yeast CaCAs.

The gate domain, composed of TM1, TM6, TM7 and TM8, and α1–5 (Fig. 1b,c), is topologically more complex, constituting a unique structure. Two long anti-parallel helices, TM1 and TM8, pack against each other and are highly tilted in the membrane (~45°). On each side near the membrane surface, this two-helix structure interacts with helical hairpins roughly parallel to the membrane. On the matrix side, two disulfide bridges stabilize a small N-terminal three-helix motif, in which α2 and α3 form a helical hairpin. On the cytosol-facing side, α4 and α5 are parallel to the membrane and interact with each other. Transmembranes 6 and 7 are short, and the loop connecting them interacts with the loop between α2 and α3 within the membrane. These four elements together constitute an expanded structure.

## Structural basis of alternating access

**Fig. 3:**
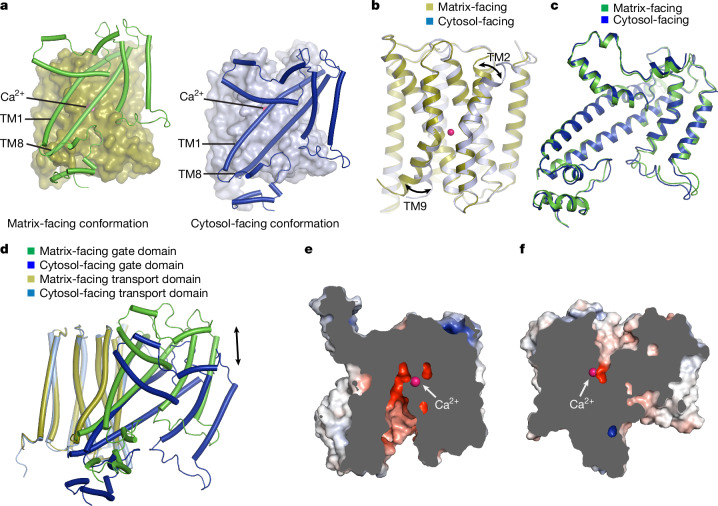
Conformational states of NCLX. **a**, NCLX in matrix- and cytosol- facing conformations. The transport domains are represented by the surface, whereas the gate domain helices are shown as cylinders. **b**, Superposition of the transport domain in matrix- and cytosol- facing conformations. **c**, Superposition of the gate domain in matrix- and cytosol- facing conformations. **d**, Superposition of NCLX in matrix- and cytosol- facing conformations. The arrow indicates the direction of the movement of the gate domain from the matrix- to the cytosol-facing conformation. **e**, The slab view of NCLX in the matrix-facing conformation. **f**, The slab view of NCLX in a cytosol-facing conformation. Ca^2+^ is shown as a magenta sphere in **e** and **f**.

We observed multiple classes of particles in distinct conformational states under both Ca^2+^-bound and -free conditions (Fig. 2c). When comparing trimers in different classes, the three transport domains superimposed well without significant conformational differences (Extended Data Fig. 5e,f), likely defining the membrane plane. By contrast, the gate domains can adopt an up (matrix-facing) or down (cytosol-facing) position relative to the transport domain. In the Ca^2+^-bound condition, we observed three distinct classes (Extended Data Fig. 3): (1) all three protomers in cytosol-facing conformation; (2) two protomers in cytosol-facing and one in matrix-facing conformation; (3) and one protomer in cytosol-facing and two in matrix-facing conformation. Under Ca^2+^-free conditions, we also observed distinct assemblies (Extended Data Figs. 2 and 4), including all three protomers in matrix-facing; one or two in cytosol-facing; or all three in cytosol-facing conformation. Comparing matrix- and cytosol-facing conformations reveals the mechanisms underlying alternating access of the substrate-binding pocket in the NCLX transport cycle (Fig. 3).

In the matrix-facing conformation, a cavity opens to the matrix and extends to near the membrane centre (Fig. 3e). This cavity is mainly lined by TM2, TM8, TM9, part of TM3, and the loop between α2 and α3. The Ca^2+^-binding site resides near the end of the cavity and is exposed to the interface between the transport and gate domains. The cavity’s negative electrostatic surface may facilitate cation transport. The highly tilted TM1 and TM8 (within the gate domain) interact with TM2 and TM9 (within the transport domain), respectively, blocking cytosolic access to the substrate translocation pathway mainly by the interacting F122^TM1^–L145^TM2^ residue pair, as well as the side chains of I433^TM8^ and N434^TM8^. Other TM1 residues also form conformation-specific interactions with residues on TM2 and TM9 to stabilize this matrix-facing conformation, including Y483^TM9^–S127^TM1^, L478^TM9^–C123^TM1^ and L144^TM2^–L126^TM1^.

In the cytosol-facing conformation, the Ca^2+^-binding site is accessible from the cytosol through a large cavity ending near the membrane centre (Fig. 3f); TM1 is packed against TM2 and TM9, with residues L114^TM1^, P152^TM2^, V441^TM8^, N467^TM9^ and F111^TM1^ coming together to seal the end of the substrate passageway. The solvent-accessible cavities in the cytosol- and matrix-facing conformations overlap near their ends. This overlapped region defines a pocket that contains the Ca^2+^-binding site, which can alternately open to either side of the membrane, supporting the alternating-access transport mechanism.

From the cytosol- to the matrix-facing conformations, both the gate and transport domains retain similar configurations with only local changes (Fig. 3b,c). The main structural transition is the relative movement (~11 Å in distance) between these two domains (Fig. 3a,d). During this drastic movement, the gate domain moves upward by ~9 Å while also shifting towards TM9 relative to TM2. Furthermore, the cytosolic side of the gate domain is brought closer to the transport domain. Accordingly, the N-terminal parts of TM2 and TM9, through which the transport domain connects with the gate domain, tilt inward and outward, respectively (Fig. 3b), resulting in a ~9 Å movement of their N-terminal ends to accommodate the large movement of the gate domain.

During the transition between cytosol- and matrix-facing states, the long, tilted anti-parallel TM1 and TM8—together with the rest of the gate domain—slide along the surface of the transport domain right next to where Ca^2+^ is bound, moving from below to above the Ca^2+^-binding site (Fig. 3a). These two transmembrane helices seal the translocation pathway from either the matrix or cytosol side, depending on NCLX’s conformation, thus functioning as a barrier that enables alternating access. In the transport domain, TM1 and TM8 interact with TM2 and TM9, where P152 and G148 (TM2), and G466 and G470 (TM9)—located mid-helix at the domain interface—surround the Ca^2+^-binding site. They form a relatively flat surface without side-chain extrusion, which may facilitate TM1 and TM8 sliding during conformational transitions. Consistently, the domain-interface residues on TM1 and TM8 are lined with hydrophobic residues, which may facilitate their movement along the transport domain.

## Ca^2+^-binding sites

**Fig. 4:**
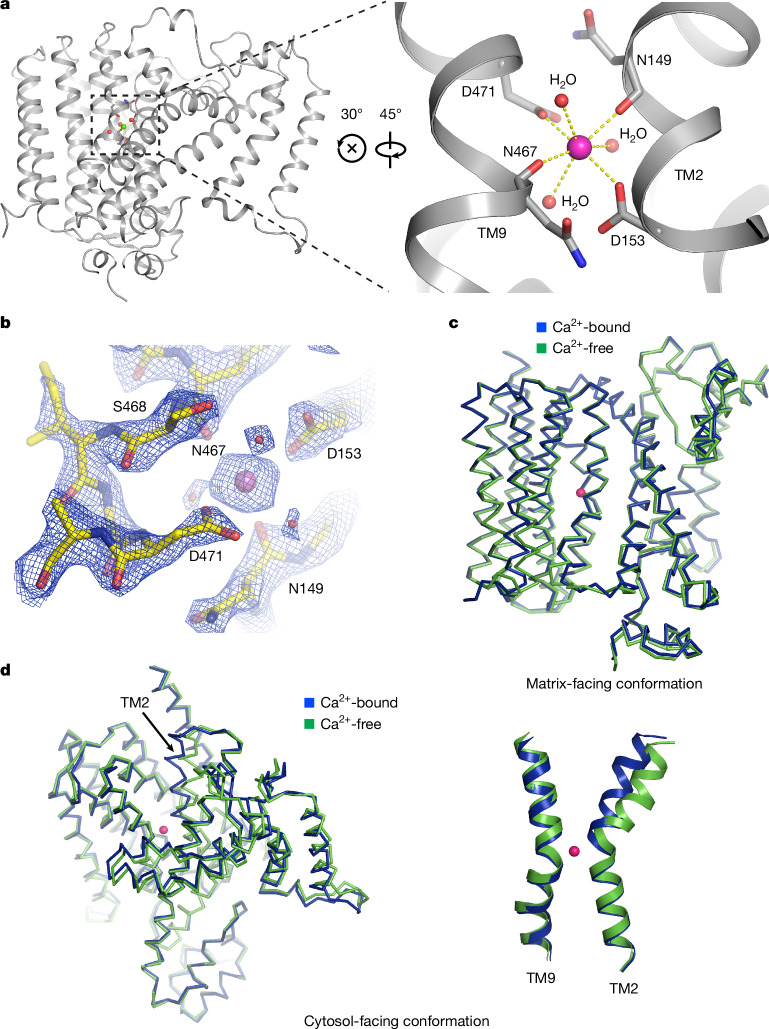
The Ca^2+^-binding site of NCLX. **a**, Coordination of Ca^2+^ at the Ca^2+^-binding site in the experimental structure (Ca^2+^ shown as a magenta sphere in the zoomed-in view on the right and as a green sphere on the left for contrast). Ca^2+^ is coordinated by the D153 and D471 side-chain oxygens; the N149 and N467 backbone oxygens; and water molecules. **b**, The density map of the Ca^2+^-binding site of NCLX. The densities are shown as blue meshes (contoured at 14*σ*), with Ca^2+^ displayed as a magenta sphere and the coordinating water molecules as red spheres. **c**, Superposition of matrix-facing NCLX in the presence and absence of Ca^2+^. **d**, Superposition of cytosol-facing NCLX in the presence and absence of Ca^2+^. A magnified view of TM2 and TM9 coordinated by Ca^2+^ is shown on the right.

The cation-binding sites of CaCA transporters are typically located between the α1 and α2 repeats^28–31,37^. With 2 mM Ca^2+^ present, a strong sphere-like non-protein density was observed in all protomers within the trimer, at a site analogous to the Ca^2+^-binding site of *Mj*NCX (Fig. 4a,b). These strong, ion-like densities were assigned to Ca^2+^, given their absence in Ca^2+^-free structures. The bound Ca^2+^ is clamped between the main-chain oxygens of N149 and N467 and the side-chains of two negatively charged residues, D153 and D471, which are conserved across NCLX homologues^27^. The high-resolution map (cytosol-open) at 2.15 Å allowed us to define the coordination geometry of Ca^2+^. The binding site adopts a classic Ca^2+^-coordination configuration with seven oxygen atoms^38^ (Fig. 4a), including the D153 and D471 side-chain oxygens; the N149 and N467 backbone oxygens; and water molecules stabilized by these residues. This seven-oxygen coordination, including carboxyl groups from acidic residues, is arranged in a (pseudo)pentagonal bipyramidal geometry—a configuration that is commonly observed in Ca^2+^-binding sites and probably underlies the Ca^2+^ selectivity of NCLX^38,39^. Furthermore, the side chains of S468 and N498, conserved across species (Extended Data Fig. 1), form hydrogen bonds with Ca^2+^-coordinating water molecules, indirectly contributing to Ca^2+^ binding (Extended Data Fig. 5g). We observed similar Ca^2+^ coordination in both cytosol- and matrix-open conformations (Extended Data Fig. 5h), indicating that the Ca^2+^ site is maintained during conformational transitions.

**Extended Data Fig. 6:**
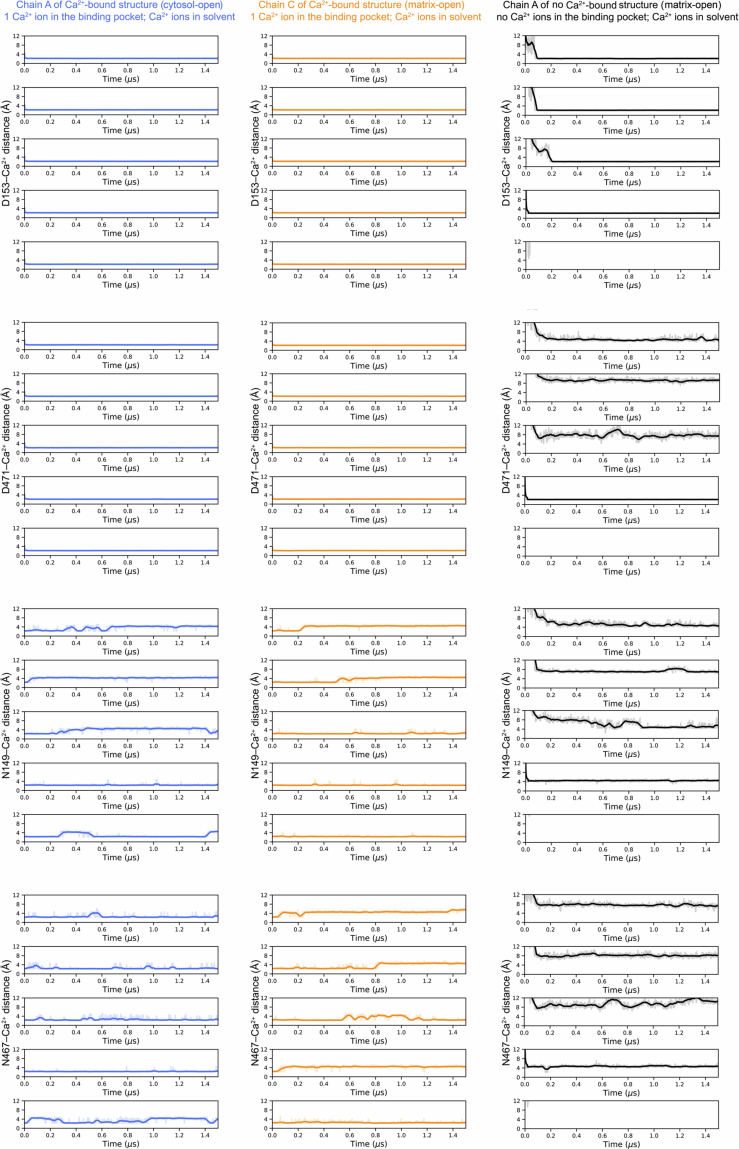
Time series of key distances in the Ca^2+^-binding site. Plots shown are for five independent simulations for each of three simulation sets: (1) simulations of cytosol-open NCLX initiated with a Ca^2+^ ion in the binding pocket (blue traces); (2) simulations of matrix-open NCLX initiated with a Ca^2+^ ion in the binding pocket (orange traces); (3) simulations of matrix-open NCLX initiated with no Ca^2+^ ions in the binding pocket (black traces). All carboxylic acidic side chains, including those of D153 and D471, are deprotonated (negatively charged) in all simulations shown. Unsmoothed traces (thin lines) and smoothed traces (thick lines) are shown for all simulations. Time traces were smoothed using a moving average with a window size of 20 ns. See Methods.

**Extended Data Fig. 7:**
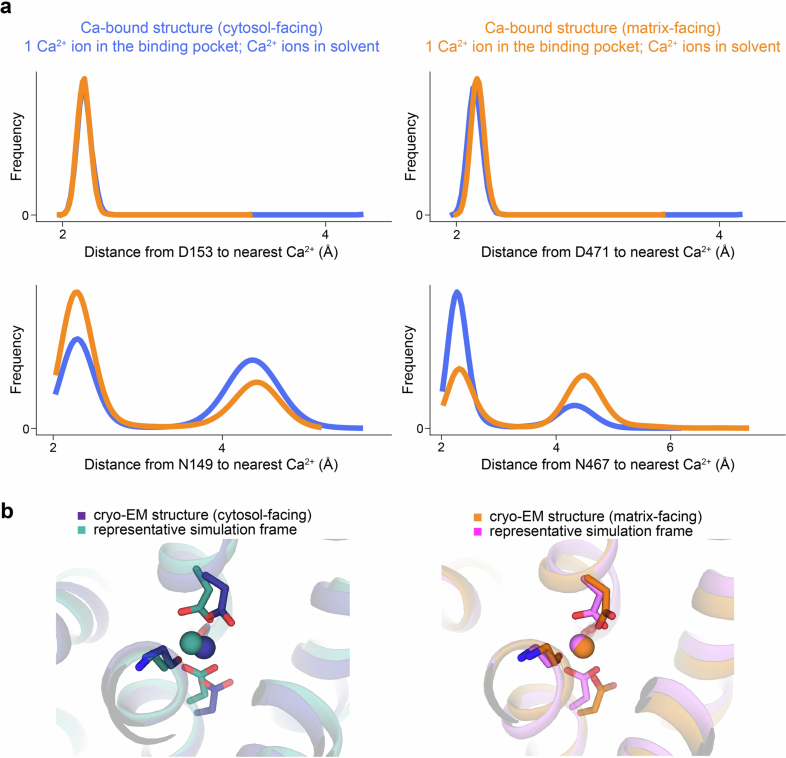
Frequency distributions of key distances in the Ca^2+^-binding site and a comparison between experimental structures and MD simulations. **a**, Distributions are computed across five independent simulations for each of two simulation sets: (left) simulations of cytosol-facing NCLX initiated with a Ca^2+^ ion in the binding pocket(blue curves); (right) simulations of matrix-facing NCLX initiated with a Ca^2+^ ion in the binding pocket (orange curves) (see Methods). All aspartate and glutamate residues are charged in all simulations shown. b, Comparison of Ca^2+^-coordinating residues in cryo-EM structures and MD simulations. Left: superposition of the cryo-EM structure of NCLX in a cytosol-facing conformation onto a representative frame from MD simulations. Right: superposition of the cryo-EM structure of NCLX in a matrix-facing conformation onto a representative frame from MD simulations.

We performed all-atom molecular dynamics simulations to further validate the Ca^2+^ site. For both matrix- and cytosol-facing conformations, simulations were initiated with a Ca^2+^ ion placed at the position identified by cryo-EM. In all such simulations (five simulations for each conformation; 2 µs each), the charged side chains of D153 and D471 form direct coordination with Ca^2+^, and the Ca^2+^ remained stably bound at this location throughout the simulations (Extended Data Fig. 6), with a similar overall coordination geometry as observed in the high-resolution experimental structure (Extended Data Fig. 7b). The coordination of Ca^2+^, including the salt bridges formed by the carboxyl oxygens of D153 and D471, is dynamic. Interestingly, in the cytosol-facing conformation, Ca^2+^ is more frequently coordinated by the N467 backbone oxygen, whereas the N149 backbone oxygen indirectly interacts through water molecules (Extended Data Fig. 7a). In comparison, in the matrix-facing conformation, Ca^2+^ is more frequently coordinated by the N149 backbone oxygen, whereas the N467 backbone oxygen indirectly interacts through water molecules. These results demonstrate the plasticity of the Ca^2+^ coordination and suggest potential differences in Ca^2+^-binding affinity according to the conformational state of NCLX.

We also initiated simulations without Ca^2+^ in the binding pocket (Extended Data Fig. 6). In the majority of these simulations, we observed Ca^2+^ diffusion into the Ca^2+^ site, initially interacting with D153 or D471. Once in this site, Ca^2+^ remained there throughout each simulation. These results further supported the Ca^2+^-binding site assigned on the basis of the cryo-EM structure.

NCLX exhibits similar overall structures with and without Ca^2+^ (both at pH 7.4) (Fig. 4c,d). In the matrix-facing conformation, the Ca^2+^-bound and -free structures superimpose well (Fig. 4c). Notably, in the cytosol-facing conformation, local conformational changes were observed in the N-terminal half of TM2, which tilts ~10° around the Ca^2+^-coordinating site (Fig. 4d). This movement brings TM2’s N terminus closer to TM9. Given that the conformational switch between cytosol- and matrix-facing states also involves tilting of the N-terminal halves of TM2 towards TM9 (Fig. 3b), these Ca^2+^-induced structural changes in the cytosol-facing conformation may potentially represent a crucial step linked to the conformational isomerization that drives substrate exchange.

## Functional characterization

Sequence alignment shows that the Na^+^-coordinating serine residues in the *Mj*NCX structure (S51 and S236 in S_int_; S77 in S_ext_) are replaced by glycine or alanine in NCLX, raising the question of how NCLX can bind three Na^+^ ions to catalyse mito-NCX, which is thought to operate in a 3:1 stoichiometry^40–42^. Structural comparison indeed reveals that NCLX lacks the Na^+^-binding sites identified in *Mj*NCX (S_ext_ and S_int_). Moreover, no obvious Na^+^ ion densities were observed in NCLX structures solved with 150 mM Na^+^ under Ca^2+^-free conditions (at the same pH of 7.4), raising the possibility that Na^+^ is a poor ligand for NCLX.

**Fig. 5:**
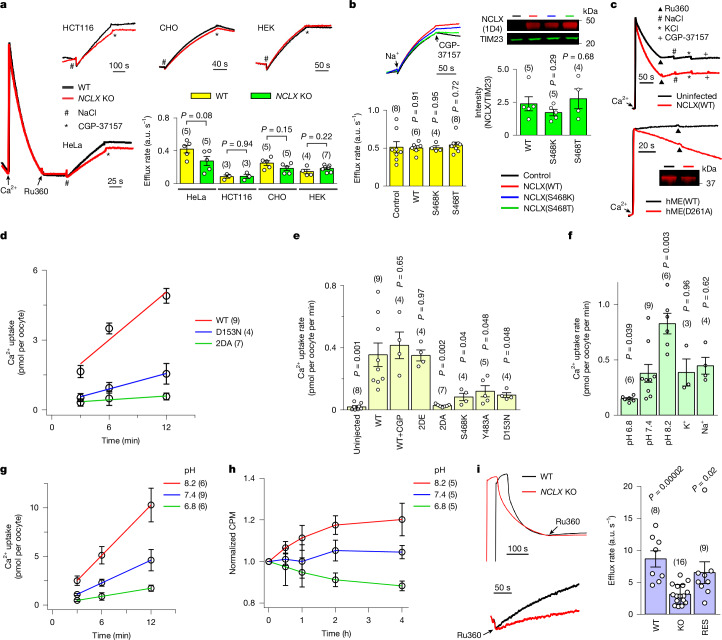
NCLX functional determination. **a**, Mito-NCX activity in indicated cell lines. Cells were digitonin-permeabilized with calcium green 5N (CG5N) for reporting extra-mitochondrial Ca^2+^. Ca^2+^ (10 µM) increases CG5N fluorescence, followed by a signal reduction reflecting mitochondrial Ca^2+^ uptake. After Ru360 inhibits Ca^2+^ uptake, 10 mM Na^+^ induces mito-NCX, abolished by 5 µM CGP-37157. Mito-NCX rates are summarized in the bar chart. **b**, Effect of expressing NCLX constructs on mito-NCX in HEK cells. Ca^2+^ efflux traces (top left), efflux rates (bottom left) and Western blots (right) compare activity and expression levels. Control: no NCLX overexpression. **c**, Mitochondrial Ca^2+^ transport in Sf9 cells. Top: 10 mM Na^+^ fails to elicit mito-NCX with or without human NCLX overexpression in permeabilized Sf9 cells. Bottom: an MCU–EMRE fusion protein (hME) with a D261A substitution induces Ru360-insensitive mitochondrial Ca^2+^ uptake. Ru360 was also added before Ca^2+^ addition to inhibit native uniporter activity. **d**, ^45^Ca^2+^ influx into *Xenopus* oocytes expressing indicated human NCLX constructs. Solid lines indicate linear fits used to obtain Ca^2+^ uptake rates. 2DA, D153A–D471A. **e**, Impact of CGP-37157 or NCLX substitutions on Ca^2+^ uptake into oocytes. Expression levels of mutants are 80–120% of WT. Uninjected, no RNA injection; 2DE, D153E–D471E. *P* values were obtained by comparing with the WT. **f**,**g**, Sensitivity of WT NCLX activity to external pH, Na^+^ (100 mM) or K^+^ (100 mM). The Ca^2+^-uptake rate summary and pH-dependence raw data are shown in **f** and **g**, respectively. Solid lines represent linear fits. **h**, H^+^-coupled Ca^2+^ flux. Oocytes expressing WT NCLX and preloaded with ^45^Ca^2+^ were exposed to the indicated external pH. Intracellular ^45^Ca^2+^ measured at various time points were normalized to the average count at *t* = 0. CPM, count per minute. **i**, NICE in HeLa cells. Following CG5N addition (initial signal jump) and digitonin-induced slow signal decline, Ru360 addition inhibits the uniporter and reveals NICE, as also shown in the zoomed-in traces (bottom left) and quantified in the bar chart. RES, NICE rescue by expressing WT NCLX in *NCLX* KO cells. Numbers in parentheses indicate independent biological repeats. The molecular mass marker unit is kilodaltons. Data show the mean ± s.e.m. Statistics was performed using an unpaired, two-tailed *t*-test (significant at *P* < 0.05). Refer to Supplementary Fig. 1 for gel source data. a.u., arbitrary unit. Source Data

To directly assess the contributions of NCLX to mito-NCX, we produced *NCLX* KO HeLa and Chinese hamster ovary (CHO) cells. Surprisingly, a standard mitochondrial Ca^2+^ flux assay (Fig. 5a) showed that, after mitochondrial Ca^2+^ uptake was abolished by a uniporter inhibitor Ru360, adding Na^+^ ions elicited Ca^2+^ efflux, which was as robust as in wild-type (WT) cells, and was strongly suppressed by a well-established mito-NCX inhibitor CGP-37157 (ref. ^43^). These results demonstrate that mito-NCX remains intact in *NCLX* KO cells. To further verify this surprising observation, we tested *NCLX* KO HEK293 and HCT116 cells that were independently generated by the Trebak laboratory^25^ and again observed strong mito-NCX (Fig. 5a).

**Extended Data Fig. 8:**
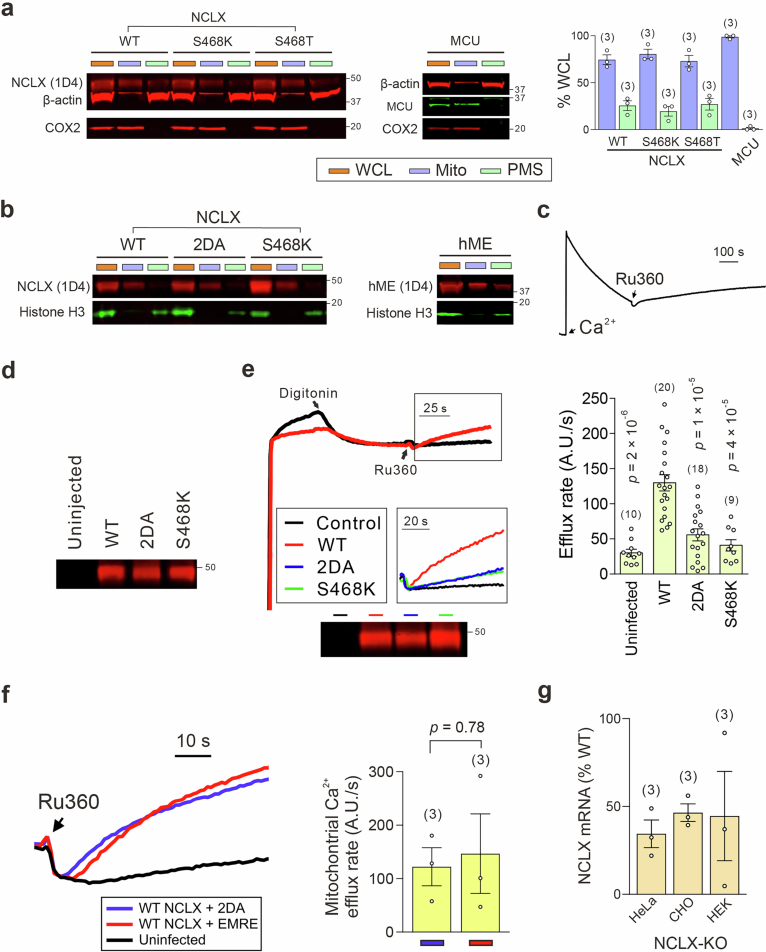
Functional analysis of NCLX. **a**, Subcellular localization of overexpressed NCLX in HEK cells. NCLX in whole-cell lysates (WCL), mitochondria (Mito), and post-mitochondrial supernatant (PMS), prepared from equal numbers of cells, was analysed. As a control to demonstrate the robustness of the subcellular fractionation, we show that MCU and a mitochondrial protein COX2 are predominantly present in the Mito fraction, while β-actin is mostly in the cytosol. Results show that overexpressed, C-terminally 1D4-tagged WT or mutant NCLX, detected by the 1D4 antibody, mostly travels to mitochondria, with only a small fraction present outside of mitochondria as summarized in the bar chart. **b**, A representative result showing the presence of NCLX in Sf9-cell mitochondria. Experiments were performed as in **a**. The absence of Histone H3 in the mitochondrial fraction confirms minimal nuclear contamination. **c**, Mitochondrial Ca^2+^ efflux in Sf9 cells. The trace supplements the upper panel in Fig. 5c to show that adding Ru360 to inhibit the uniporter reveals mitochondrial Ca^2+^ efflux in Sf9 cells. **d**, NCLX in *Xenopus* oocyte membranes. The plasma membranes of *Xenopus* oocytes expressing 1D4-tagged WT or mutant NCLX were isolated, as described in Materials and Methods, and were analysed with Western blot using the 1D4 antibody. **e**, NCLX-induced NICE in Sf9 mitochondria. WT, 2DA (D153A-D471A), or S468K NCLX was expressed to similar levels in sf9 cells, as shown in the Western blot image. Mitochondrial Ca^2+^ flux experiment, performed in the absence of Na^+^, shows that blocking the uniporter with Ru360 reveals NICE mediated by NCLX. The rate of NICE in Sf9 mitochondria is ~10-fold faster than that in HeLa cells (Fig. 5i), likely because higher NCLX expression in Sf9 cells. The p value in the bar chart was obtained by comparing with WT NCLX. Control: uninfected Sf9 cells. **f**, The effect of functionally impaired NCLX mutants on WT NCLX. WT NCLX was co-expressed with 2DA NCLX or an unrelated protein EMRE as a control in Sf9 cells. 2DA NCLX does not affect NICE mediated by WT NCLX, suggesting that a functionally impaired mutant would not affect the function of WT subunits in the NCLX trimer. **g**, NCLX mRNA levels in various NCLX-KO cell lines. The bar chart presents NCLX mRNA levels in NCLX-KO cell lines as compared with their corresponding WT cells. Note that CRISPR KO would not eliminate NCLX mRNA, because it works by introducing insertion or deletion into the NCLX gene to cause frameshifts, instead of targeting mRNA for degradation as in the case of shRNA. Throughout the entire figure, data are presented as means ± s.e.m., with values in parentheses denoting the number of independent biological replicates, and statistics performed using unpaired, two-tailed t-test. Experiments in panels b-d were performed with three independent biological replicates with consistent results. Molecular weight marker unit: kDa. For gel source data, see Supplementary Fig. 1. Source Data

We then overexpressed either NCLX(WT) or an NCLX(S468T) mutant previously proposed to be dominant-negative^1^. Both proteins properly localized to mitochondria (Extended Data Fig. 8a), but neither affected mito-NCX (Fig. 5b). Interestingly, insect Sf9 cell lines showed rapid mitochondrial Ca^2+^ uptake but lacked mito-NCX activity (Fig. 5c). Expressing human uniporter subunits MCU (a Ru360-insensitive variant) and EMRE in Sf9 cells produced Ru360-insensitive mitochondrial Ca^2+^ uptake (Fig. 5c). By contrast, expressing similar levels of human NCLX in Sf9 mitochondria (Extended Data Fig. 8b) failed to yield mito-NCX (Fig. 5c and Extended Data Fig. 8c). Altogether, these mitochondrial Ca^2+^ flux experiments (Fig. 5a–c), along with sequence and structural analyses, strongly suggest that NCLX does not mediate mito-NCX.

To establish an assay for studying NCLX function, we first attempted to reconstitute rat NCLX into liposomes; however, the proteoliposomes exhibited a large non-specific Ca^2+^ leak, making it impossible to isolate NCLX-specific signals. This leak probably stems from the lauryl maltose neopentyl glycol (LMNG) detergent, which is used to purify NCLX but is difficult to remove from liposomes due to its low critical micelle concentration. We then turned to the *Xenopus* oocyte expression system, widely employed to study NCX proteins^44–46^. A fraction of human NCLX was directed to the oocyte plasma membrane through codon optimization and targeting sequence modification (Extended Data Fig. 8d). As in the initial characterization of NCX1 (ref. ^44^), we quantified NCLX activity by measuring ^45^Ca^2+^ influx into oocytes. Results show that NCLX exhibited robust Ca^2+^ transport activity, which was absent in oocytes lacking heterologous NCLX expression (Fig. 5d,e). This Ca^2+^ transport was unaffected by CGP-37157 but was strongly suppressed by (1) mutating NCLX’s Ca^2+^-coordinating residues D153 and D471; (2) Ala substitution of Y483, a critical residue that seals the cytoplasmic entrance in the matrix-facing conformation; or (3) introducing a positive charge into the Ca^2+^-binding pocket via an S468K substitution (Fig. 5d,e). These results demonstrate that NCLX is a Ca^2+^ transporter and highlight the functional importance of critical NCLX residues observed in the structures.

If NCLX is an exchanger like other CaCA proteins, its Ca^2+^ transport should be sensitive to the electrochemical gradients of coupled ions. Imposing inward Na^+^ or K^+^ gradients across the oocyte membrane did not affect NCLX Ca^2+^ transport (Fig. 5f); however, Ca^2+^ transport was enhanced by increasing the external pH and then suppressed by acidification (Fig. 5f,g), suggesting potential H^+^ coupling. A H^+^/Ca^2+^ exchange mechanism demands that NCLX uses a H^+^ gradient to drive energetically uphill movement of Ca^2+^. Indeed, reducing external pH in NCLX-expressing oocytes—preloaded with ^45^Ca^2+^—causes Ca^2+^ efflux against the gradient (Fig. 5h), establishing NCLX as a H^+^/Ca^2+^ exchanger.

It has long been recognized that mitochondria can extrude matrix Ca^2+^ via Na^+^-independent H^+^/Ca^2+^ exchange (NICE)^17^. Consistent with NCLX being a H^+^/Ca^2+^ exchanger, *NCLX* KO reduced the rate of NICE in HeLa cells—an effect restored by expressing WT NCLX (Fig. 5i). Moreover, WT NCLX expression in Sf9 cells induced robust NICE, whereas functionally impaired NCLX mutants—NCLX(D153A–D471A) or NCLX(S468K)—elicited minimal NICE (Extended Data Fig. 8e,f). Altogether, these mitochondrial Ca^2+^ flux assays further support our proposed NCLX function.

**Extended Data Fig. 9:**
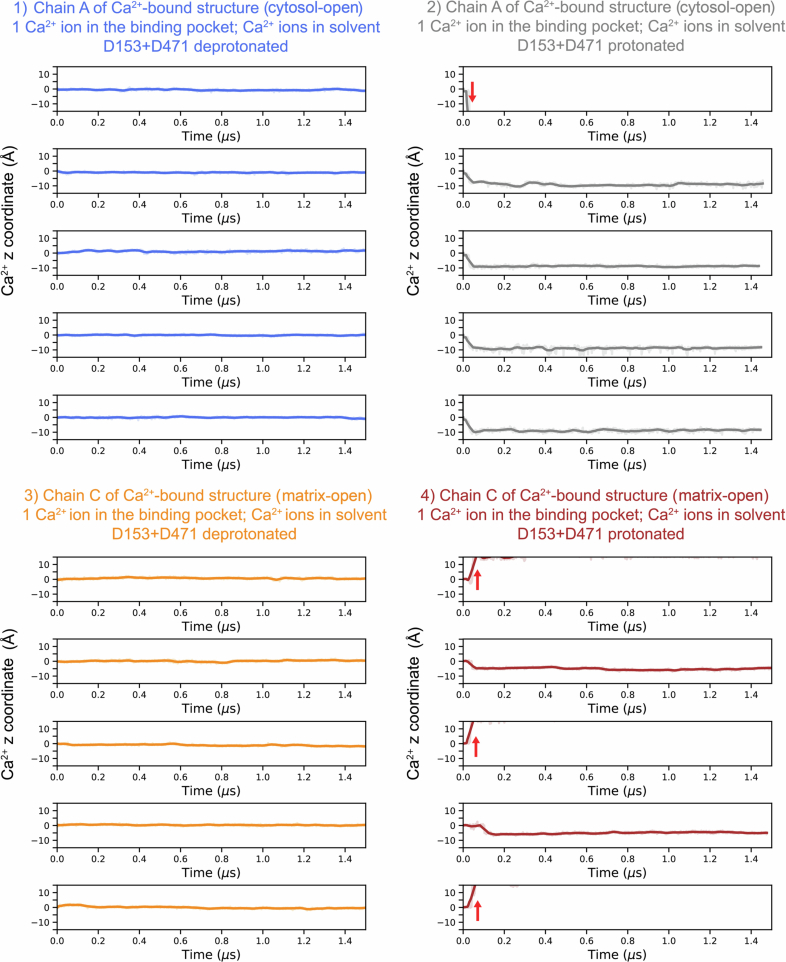
Time series of Ca^2+^ position in simulations with D153 and D471 deprotonated or protonated. Plots are shown for five independent simulations under each of four conditions: (1) simulations of cytosol-open NCLX initiated with a Ca^2+^ ion in the binding pocket, with D153 and D471 deprotonated (blue traces); (2) simulations of cytosol-open NCLX initiated with a Ca^2+^ ion in the binding pocket, with D153 and D471 protonated(gray traces); (3) simulations of matrix-open NCLX initiated with a Ca^2+^ ion in the binding pocket, with D153 and D471 deprotonated (orange traces); (4) simulations of matrix-open NCLX initiated with a Ca^2+^ ion in the binding pocket, with D153 and D471 protonated (brown traces). The z-axis is the direction perpendicular to the lipid bilayer. The z-coordinate is 0 at the initial Ca^2+^ position, with values increasing in the direction of the matrix and decreasing in the direction of the cytosol. Red arrows mark times at which the Ca^2+^ exits the transporter; subsequently, it either interacts with charged residues at the protein surface or diffuses freely through the solvent. Unsmoothed traces (thin lines) and smoothed traces (thick lines) are shown for all simulations. Time traces were smoothed using a moving average with a window size of 20 ns.

As our structural, functional and molecular dynamics simulation data all suggest that D153 and D471 are critical for Ca^2+^-binding, we propose that H^+^/Ca^2+^ exchange occurs when H^+^ binds to the side chains of D153 and/or D471, reducing Ca^2+^ affinity to cause Ca^2+^ release. This mechanism is analogous to those proposed for the *Saccharomyces* *cerevisiae* H^+^/Ca^2+^ exchanger Vcx1 and *Archaeoglobus fulgidus* H^+^/Ca^2+^ exchanger *Af*CAX (refs. ^29,30^), and is consistent with the *Mj*NCX structure solved at a low pH of 4, where protonation of E54 and E213—corresponding to NCLX(D153) and NCLX(D471)—prevents Ca^2+^ or Na^+^ binding^37^. Indeed, a D153N substitution reduced NCLX Ca^2+^ transport activity, presumably due to compromised Ca^2+^-binding (Fig. 5d,e; the NCLX(D471N) mutant was not analysed due to much lower expression). By contrast, a double D153E–D471E substitution, which preserves side-chain charges, retained transport function (Fig. 5e). Furthermore, molecular dynamics simulations revealed that protonation of D153 and D471 led to a decrease in stability of the bound Ca^2+^, with Ca^2+^ exiting the transporter entirely in several of these simulations (Extended Data Fig. 9), providing further support for the proposed H^+^/Ca^2+^ exchange mechanism.

## Discussion

Here we elucidated the structural and functional mechanisms of the key mitochondrial transporter NCLX. Structural analyses of oligomer formation and substantial vertical movement of the gate domain in the membrane reveal an intriguing parallel to transporters that operate via an elevator alternating-access mechanism^47^, including the sodium–aspartate co-transporter Glt_Ph_ (ref. ^48^), sodium–proton antiporter NhaA (ref. ^49^), bile acid transporters^50,51^ and sodium–nucleoside co-transporter CNT (ref. ^52^). In both NCLX and elevator transporters, the substrate-binding site resides in the transport domain, whereas a pair of tilted anti-parallel transmembrane helices separate the solvent from either side of the membrane. The substrate-binding site becomes either above or below the barrier formed by these two transmembrane helices during an elevator-like movement of one domain relative to the other, thus giving rise to alternating access.

However, NCLX seems to fundamentally differ from elevator transporters. First, in NCLX, the transport domain mediates oligomerization, whereas this interaction is provided by the scaffold domain in elevator transporters. Second, whereas the transport domain moves considerably to carry substrates across the membrane in elevator transporters, the transport domain and substrate-binding site in NCLX seem to remain stationary during conformational transitions (C_α_ r.m.s.d. = 0.54 Å for the transport domain, excluding the N-terminal part of TM2 and TM9 that connect to the gate domain). NCLX seems to use an ‘elevator-gate’ that traverses the membrane to control alternating access. This distinct transport mechanism may give rise to relative movement between the transport domain and the domain that contains the hydrophobic barrier for alternating access. Notably, the bacterial CAX transporter YfkE also adopts a trimeric architecture, with the transport domain mediating trimer formation and the hydrophobic barrier portion located on the periphery^31^. This hints that its transport domains, like NCLX, remain stationary in the membrane.

Some other families of CaCA transporters were thought to operate via a rocker-switch mechanism^47^, which is distinct from the elevator-gate mechanism of NCLX. Interestingly, comparing inward- and outward-facing conformations of those CaCA transporters from different families^28–32^, along with molecular dynamics simulations of archaeal *Mj*NCX (ref. ^33^), suggests similarly large movements of the transmembrane regions equivalent to NCLX’s TM1–TM8 pair. For those CaCA transporters, it is unclear whether the TM1–TM8 pair constitutes a separate structural domain and how these transporters move within the membrane without oligomerization to define the stationary part. Nonetheless, those structural comparisons and simulations suggest a critical role for TM1–TM8 in the transport cycle, lending support to the conformational changes observed within a protomer of NCLX.

The CaCA protein superfamily contains transporters that exchange Ca^2+^ with various cations, including H^+^, Na^+^ or K^+^. A key finding in this work is that NCLX functions as a H^+^/Ca^2+^ exchanger rather than a Na^+^/Ca^2+^ exchanger. Indeed, phylogenetic analysis suggests that NCLX is distant from canonical NCX proteins and more closely related to CCX-family proteins in plants^27^. The exact exchange mechanism for these plant proteins is unclear, but they also lack multiple Na^+^-coordinating residues in *Mj*NCX, suggesting that Na^+^ is unlikely to be the coupled ion for Ca^2+^ transport. A H^+^/Ca^2+^ exchange mechanism similar to that in NCLX would seem more plausible, given that pH gradients—not Na^+^ gradients—are the primary driving force for secondary active transport in plants^53^.

Our structure determined at a low pH of 5.5 without Ca^2+^ presumably represents proton-loaded NCLX, given the ~100-fold higher proton concentration compared with the transporter’s typical operating conditions. Notably, under this condition, the structure exhibits cytosol-open conformations across all protomers within a trimer, suggesting that this conformation is predominant at low pH. By contrast, the structure determined at pH 7.4 without Ca^2+^ displays a combination of cytosol- and matrix-open, or all matrix-open, conformations within a trimer, compatible with NCLX potentially in mixed protonated states. This pH-dependent shift in conformational state distribution aligns with our finding that NCLX operates a H^+^-coupled transport process. Furthermore, we found that, for each protomer, the overall structure of this putative proton-bound NCLX is similar to the Ca^2+^-bound form, with the N-terminal half of TM2 rotating around the Ca^2+^-binding site along the interface with the gate domain (Extended Data Fig. 5i). This rotation is presumably caused by local changes in the Ca^2+^-binding site configuration, whereas the domain-interface residues of TM2 remain staying on the interface. It is conceivable that these local conformational differences may potentially influence key steps in a transport cycle. Together, our Ca^2+^-bound and putative proton-loaded structures provide a structural framework for dissecting NCLX transport. The exact stoichiometry and exchange mechanism still require future investigation.

For decades, excitable cells have been known to exhibit much stronger mito-NCX than non-excitable cells^54^; however, after the identification of NCLX, its expression was found to be homogeneous across tissues^55,56^. This discrepancy can now be resolved in light of our finding that NCLX does not mediate mito-NCX. We note that our results do not contradict most previous studies analysing how *NCLX* KO impacts mitochondrial Ca^2+^ transport^4,6,19,22,25,35,36^. In particular, most studies report that in intact cells, loss of NCLX reduces the rate of mitochondrial Ca^2+^ efflux to varying degrees. However, this reduction may reflect a decrease in NICE, to which NCLX contributes (Fig. 5i), or may arise indirectly from altered Ca^2+^ electrochemical gradients following *NCLX* KO. Our results are not inconsistent with previous efforts to identify mitochondrial H^+^/Ca^2+^ exchangers^57–60^. In our hands, *NCLX* KO does not fully abolish NICE in HeLa cells, raising the possibility that more than one transporter might underlie mitochondrial NICE.

There is no doubt that NCLX is critical for normal physiology, as evidenced by the wide range of phenotypes and pathologies induced by NCLX ablation; however, how exactly NCLX regulates mitochondrial processes must now be understood in the context of its role in H^+^/Ca^2+^ rather than Na^+^/Ca^2+^ exchange. In mitochondria, the proton motive force across the IMM, produced by proton pumping via the electron transport chain, is harvested by the F_o_F_1_-ATPase to synthesize ATP. Moreover, matrix [Ca^2+^] regulates oxidative phosphorylation by modulating the activity of TCA cycle enzymes. Thus, a H^+^/Ca^2+^ exchange machinery linking the proton motive force to mitochondrial Ca^2+^ homeostasis could create a feedback mechanism to regulate mitochondrial bioenergetics. Such a scenario might help to explain why *NCLX* KO often causes severe metabolic phenotypes. Clearly, further investigation is needed to link NCLX’s molecular properties to its critical roles in health and disease.

## Methods

### Protein expression and purification

The rat NCLX gene with a Strep tag at the C terminus was cloned to the BacMam vector^61^. The NCLX virus was generated and used to infect HEK293 cells at a cell density of 3 × 10^6^ cells per millilitre at 37 °C. After 12 h, the cells were cultured in the presence of 10 mM sodium butyrate at 30 °C. After another 48 h, cells were harvested and stored at −80 °C for further use.

To purify the NCLX protein under the high-Ca^2+^ condition (2 mM Ca^2+^), the cells were dounce-homogenized in buffer A (50 mM HEPES pH 7.4, 150 mM NaCl, 2 mM CaCl_2_) with protease inhibitors and ribonuclease. The membranes were solubilized at 4 °C for 2 h with the addition of 1.5% LMNG (Anatrace) and cholesteryl hemisuccinate (Anatrace) mixture. Following spinning at 18,000 r.p.m. for 45 min, the supernatant was mixed with Strep-Tactin resin (IBA) and incubated for 2 h at 4 °C. After extensive washing using buffer B (150 mM NaCl, 2 mM CaCl_2,_ 20 mM HEPES pH 7.4) containing 0.05% glyco-diosgenin (GDN, Anatrace), the protein was eluted using buffer B with 0.01% GDN and 10 mM desthiobiotin. To polish the protein, the eluted sample was concentrated and subjected to further purification using a Superose 6 increase column (Cytiva) in buffer B containing 0.006% GDN. The peak fractions of NCLX were concentrated to 15 mg ml^–1^ for preparing cryo-EM grids. To purify the NCLX protein in a Ca^2+^-free state at pH 7.4, the 2 mM CaCl_2_ was replaced with 5 mM EGTA. To purify the protein in a calcium-free state at low pH, the protein was initially extracted in the same Ca^2+^-free pH 7.4 conditions as above and gradually exchanged into buffer C (20 mM buffer with sodium acetate-acetic acid pH 5.5, 150 mM NaCl, 5 mM EGTA) containing 0.006% GDN for preparing cryo-EM grids.

### Cryo-EM sample preparation and data collection

Protein sample (3 µl) was used for each cryo-EM grid. The glow discharged grids (300 mesh, R2/1, Au, holey carbon, Quantifoil) with the sample were blotted using a Vitrobot Mark IV for 3 s (4 °C and 100% humidity) or a Leica EM GP2 for 2 s (4 °C and 96% humidity), before being plunge frozen in liquid ethane.

For the NCLX sample without calcium (pH 7.4), datasets were collected on Titan Krios (300 keV; with GIF-Quantum Energy Filter) at cEMc/Stanford or S2C2/Stanford. Images were recorded using K3 Summit detector (Gatan) (super-resolution counting mode; 105,000× magnification; 0.86 Å physical pixel size) by serialEM. The Multiple Record method was used for data collection (image shift; 3 × 3). A total of 50 video frames were collected.

For the NCLX sample with calcium, and the NCLX low-pH sample without calcium, datasets were acquired using EPU software, similarly on Titan Krios (cEMc/Stanford or S2C2/Stanford) with Falcon 4 detector (Thermo Fisher Scientific; counted mode) at 96,000× nominal magnification (0.82 Å physical pixel size) or 130,000× nominal magnification (0.946 Å or 0.95 Å physical pixel size). Forty movie frames were collected in MRC format, with a dose of ~50 electrons per Å^2^.

### Cryo-EM data processing

For NCLX without calcium data (pH 7.4), a total of 4,264 movies were processed by cryoSPARC live^62^ v4.2 (Patch motion correction, Patch CTF estimation and template picking). Then, 821,498 particles were extracted with 80-pixel box and 4 × 4 binning from 3,241 images (with <8 Å CTF estimates). Heterogeneous refinement was performed over three rounds, using accurate and biased maps. This resulted in 95,304 particles for subsequent steps. Furthermore, 57,247 particles from 1,304 images were selected for Topaz (v.0.2.4)^63^ training. Topaz repicking subsequently yielded 659,059 particles (extracted with 80-pixel box and 4 × 4 binning), which underwent 3D classification using a seed-facilitated multi-reference strategy^64^. The reference sets included (1) non-uniform refinement accurate map; (2) maps with a resolution gradient (an accurate map along with low-pass filtered maps at 10 Å and 20 Å); (3) biased maps via ab initio reconstruction; and (4) maps with noise re-weight (accurate map and maps with micelles scaled down by 0.3 and 0.7). After removing duplicated particles, 208,064 particles were re-extracted with a 320-pixel box. Two rounds of heterogeneous refinement were performed with noise reweighted and noise accurate maps, respectively. Afterwards, 86,447 selected particles gave rise to a 3.29 Å map (class 1) through successive rounds of non-uniform, Local CTF and Local refinements. The map was sharpened by DeepEMhancer^65^. Similarly, 34,185 particles underwent non-uniform refinement, yielding a 3.93 Å map (class 2) and 26,871 particles yielded a 4.29 Å map (class 3). The local resolution estimate was performed by BlocRes.

For NCLX with calcium data, datasets A and B were collected and processed via the same processing strategy; thus, dataset A is described in the following steps. For dataset A, 13,255 videos were motion corrected using MotionCorr2 (ref. ^66^). CryoSPARC (v.4.2) was used to process the dose-weighted images, including Patch CTF estimation. Topaz picking resulted in 3,180,107 particles (80-pixel box and 4 × 4 binning) from 12,725 images (with <8 Å CTF estimates). Heterogeneous refinement was performed over three rounds with accurate and biased maps. This resulted in 390,406 seed particles for subsequent processing. Furthermore, the previous 3,180,107 particles underwent 3D classification using the seed-facilitated multi-reference strategy with similar multiple-references as described above. After removing duplicates, the resulting 948,917 particles were combined with the seed particles, yielding 1,021,442 particles with duplicate removal. Two rounds of heterogeneous refinement were performed using noise reweighted maps. Afterwards, the re-extracted particles (with 320-pixel box) were subjected to two rounds of heterogeneous refinement with noise reweighted and accurate maps, respectively. The outcome was two classes with 334,585 particles and 191,077 particles, which were combined with 155,219 and 230,218 particles from dataset B using the same processing strategy as dataset A, respectively. The combined particles underwent heterogeneous, non-uniform, local CTF and non-uniform refinements. Refinement of a high-quality subset of 150,233 particles then yielded a 3.31 Å map (class 3a), and 145,777 particles yielded a 2.97 Å map (class 2a). The selected 132,072 particles were refined, yielding a 3.10 Å map (class 4a) after 265,702 particles underwent 3D classification without alignment. All maps were sharpened by DeepEMhancer for improved density. For dataset C, 10,373 videos were motion corrected using MotionCorr2. The dose-weighted images were imported and estimated by Patch CTF estimation using cryoSPARC v4.4. After Blob picking from 500 images, 158,465 selected particles were subjected to 2D classification and ab initio refinement, yielding 97,913 particles as seeds. Simultaneously, following template picking for whole images, 9,112,846 particles (extracted with 80-pixel box, 4 × 4 binning) underwent 3D classification using seed-facilitated multi-references. After duplicate removal, 1,423,457 re-extracted particles (with 320-pixel box) underwent three rounds of heterogeneous refinement (one with accurate and biased maps, followed by two with accurate map). The outcome was two classes with 686,084 and 205,206 particles, respectively. Refinement of the selected subset of 686,084 particles then yielded a 2.15 Å map with C3 symmetry (class 4a). The other 205,206 particles were processed via heterogeneous refinement, yielding 115,207 particles, which were further refined to produce a 2.60 Å map (class 3a). Classes 3a and 4a exhibit higher resolution compared to those in datasets A and B. Consequently, these higher-resolution maps derived from dataset C are retained for use in the paper. The local resolution estimate was performed using BlocRes in cryoSRARC.

For NCLX at low-pH (without calcium) sample, a total of 9,653 videos were imported into cryoSPARC live v4.4 and processed through steps similar to those described above for the NCLX dataset without calcium at pH 7.4, including particle extraction with 80-pixel box and 4 × 4 binning. This resulted in 5,574,228 particles from 9,593 images with CTF estimate better than 8 Å. Three-dimensional classification using the seed-facilitated multi-reference strategy yielded 816,107 particles, following duplicate removal. In parallel, following topaz picking, 2,221,595 particles underwent 2D classification (two rounds) and heterogeneous refinement (three rounds), resulting in 115,569 particles as seeds. These 2,221,595 particles underwent 3D classification using the seed-facilitated multi-reference strategy, which resulted in 805,025 particles after duplicate removal. These two sets of particles were combined, resulting in 1,117,206 particles after duplicate removal. Following one round of heterogeneous refinement using noised-reweighted map, 683,191 particles were re-extracted with 320-pixel box. Finally, these particles were refined with C_3_ symmetry, yielding a 2.62 Å map (class 4b). The local-resolution estimate was performed using BlocRes in cryoSRARC.

### Model building and refinement

The initial model used for NCLX structure was obtained through AlphaFold2^67^ prediction. Chimera was then used to fit the initial model into the cryo-EM map, which was manually rebuilt using Coot^68^. The rebuilt model was then subjected to refinement in Phenix^69^ to optimize its geometry and stereochemistry and was assessed by MolProbity. The two structures, including class 1 from NCLX without calcium, and class 2a from NCLX with calcium, were manually adjusted and refined, the other structures used classes 1 and 2a as initial model and were manually adjusted and fitted into the corresponding map. Compared with other classes, density maps for classes 2 and 3 (in the Ca^2+^-free, pH 7.4 condition) are at lower resolution. In these two classes, side chains were removed in regions where the corresponding maps lack sufficient information, and their register was based on corresponding models in the Ca^2+^ bound forms. Figures were generated using PyMOL (Schrödinger)^70^, UCSF Chimera^71^ and ChimeraX^72^.

### System set-up for molecular dynamics simulations

We performed simulations of NCLX under five conditions: (1) simulations of cytosol-open NCLX initiated with a Ca^2+^ ion in the binding pocket, with residues D153 and D471 deprotonated; (2) simulations of matrix-open NCLX initiated with a Ca^2+^ ion in the binding pocket and residues D153 + D471 deprotonated; (3) simulations of matrix-open NCLX initiated with no Ca^2+^ ion in the binding pocket and residues D153 + D471 deprotonated; (4) simulations of cytosol-open NCLX initiated with a Ca^2+^ ion in the binding pocket and residues D153 + D471 protonated (neutral); and (5) simulations of matrix-open NCLX initiated with a Ca^2+^ ion in the binding pocket and residues D153 + D471 protonated. We initiated all simulations from the cryo-EM structures of NCLX (based on an earlier version of the cryo-EM maps): simulation sets (1) and (4) were initiated using chain A of the Ca^2+^-bound NCLX structure; simulation sets (2) and (5) were initiated using chain C of the Ca^2+^-bound NCLX structure; and simulation set (3) was initiated using the chain A of the NCLX structure with no Ca^2+^ bound. The Ca^2+^ ion in the Ca^2+^-bound NCLX structure was preserved for simulation sets (1), (2), (4) and (5). We performed five independent simulations for each simulation condition, each at least 1.4 µs in length. Initial atom velocities were assigned randomly and independently for each simulation.

For all simulation conditions, the protein structure was aligned with the Orientations of Proteins in Membranes^73^ entry for 3V5U (NCX from *Methanocaldococcus jannaschii*^28^) using PyMOL, and water molecules from 3V5U were incorporated. Prime (Schrödinger)^74^ was used to add capping groups to protein chain’s termini. Protonation states of all titratable residues other than D153 and D471 were assigned at pH 7. Histidine residues were modelled as neutral, with a hydrogen atom bound to either the delta or epsilon nitrogen depending on which tautomeric state optimized the local hydrogen-bonding network. Using Dabble^75^, the prepared protein structures were inserted into a pre-equilibrated palmitoyl-oleoyl-phosphatidylcholine bilayer, the system was solvated, and calcium and chloride ions were added to neutralize each system at a calcium concentration of 75 mM and a chloride concentration of 150 mM. The final systems comprised approximately 135,000 atoms, and system dimensions were approximately 120 × 140 × 110 Å (Supplementary Table 2).

### Molecular dynamics simulation and analysis protocols

The simulation protocol was similar to that used in previous work^76^, as those simulations proved sufficient to describe atomic-level interactions between a membrane transporter and its substrate. We used the CHARMM36m force-field for proteins, the CHARMM36 force-field for lipids and ions, and the TIP3P model for water molecules^77–79^. All simulations were performed using the Compute Unified Device Architecture version of particle-mesh Ewald molecular dynamics in AMBER20 on graphics processing units.

Systems were first minimized using three rounds of minimization, each consisting of 500 cycles of steepest descent followed by 500 cycles of conjugate gradient optimization. Harmonic restraints (10.0 and 5.0 kcal mol^−1^ Å^−2^) were applied to the protein and lipids for the first and second rounds of minimization, respectively. Then, 1 kcal mol^−1^ Å^−2^ harmonic restraints were applied to the protein for the third round of minimization. Systems were then heated from 0 K to 100 K in the NVT ensemble over 12.5 ps, and then from 100 K to 310 K in the NPT ensemble over 125 ps, using 10.0 kcal mol^−1^ Å^−2^ harmonic restraints applied to protein heavy atoms. Subsequently, systems were equilibrated at 310 K and 1 bar in the NPT ensemble, with harmonic restraints on the protein non-hydrogen atoms tapered off by 1.0 kcal mol^−1^ Å^−2^ starting at 5.0 kcal mol^−1^ Å^−2^ in a stepwise fashion every 2 ns for 10 ns, and then by 0.1 kcal mol^−1^ Å^−2^ every 2 ns for 20 ns. Production simulations were performed without restraints at 310 K and 1 bar in the NPT ensemble using the Langevin thermostat and the Monte Carlo barostat, and using a timestep of 4.0 fs with hydrogen mass repartitioning^80^. Bond lengths were constrained using the SHAKE algorithm^81^. Non-bonded interactions were cut off at 9.0 Å, and long-range electrostatic interactions were calculated using the particle-mesh Ewald method with an Ewald coefficient of approximately 0.31 Å^−1^, and fourth-order B-splines. The particle-mesh Ewald grid size was chosen such that the width of a grid cell was approximately 1 Å. Trajectory frames were saved every 200 ps during the production simulations. The AmberTools17 CPPTRAJ package was used to reimage trajectories^82^. Simulations were visualized and analysed using Visual Molecular Dynamics (VMD)^83^ and PyMOL^70^. The D153–Ca^2+^ distance is the minimum distance between a side-chain oxygen of D153 and a Ca^2+^ ion. The D471–Ca^2+^ distance is the minimum distance between a side-chain oxygen of D471 and a Ca^2+^ ion. The N467–Ca^2+^ distance is the minimum distance between the backbone oxygen of N467 and a Ca^2+^ ion. The N149–Ca^2+^ distance is the minimum distance between the backbone oxygen of N149 and a Ca^2+^ ion. In Extended Data Fig. 7a, to construct the probability distributions for these distance metrics, we used trajectory frames from all simulations under each condition and applied a Gaussian kernel density estimator.

### Proteomic sample preparation for mass spectrometry analysis

The mass spectrometry (MS) sample preparation, data collection and data analyses follow similar protocols to those previously reported^84^. To prepare NCLX for liquid chromatography–tandem mass spectrometry (LC–MS/MS) analysis, human NCLX with a C-terminal Strep-tag II was cloned into the BacMam vector and expressed and purified as described above for rat NCLX. Purified NCLX was then processed for proteomics analysis using an S-Trap Micro Column (C02-micro-80, ≤100 µg, Protifi). Briefly, purified proteins were reduced by incubating with 10 mM dithiothreitol at 95 °C for 10 min and subsequently alkylated using 40 mM 2-chloroacetamide at room temperature for 60 min. Phosphoric acid (final concentration = 1.2%) was added to acidify the denatured, non-digested proteins before loading the samples onto S-trap columns. The columns were washed three times with 100 mM triethylammonium bicarbonate (TEAB) in 90% methanol. To digest the bound proteins, each column was incubated with Trypsin (Promega, V5113) in 50 mM TEAB at a 1:20 trypsin-to-substrate ratio (w/w) at 37 °C overnight. Elutions were performed in succession using 50 mM TEAB (40 μl), 0.2% formic acid (FA) (40 μl), and then 50% acetonitrile (ACN) + 0.2% FA (40 μl) to elute the peptides from the column. The combined eluate was dried using a vacuum concentrator and resuspended in 10 μl of 0.2% FA, 3% ACN.

To analyse the presence of NCLX in WT and KO HeLa cells, the mitochondrial fraction was enriched by resuspending cells in mitochondrial resuspension buffer (5 mM HEPES-KOH pH 7.2, 250 mM sucrose). Cells were lysed using dounce homogenization, and large cellular debris were pelleted by centrifuging the lysate at 1,500 g for 10 min. The supernatants were then collected and further centrifuged at 13,000 g for 10 min, and the pellet was resuspended in mitochondrial resuspension buffer. This resuspension was then centrifuged at 13,000 g for 10 min to pellet mitochondria. To solubilize NCLX, the mitochondria were incubated with 1.5% m/v LMNG/cholesteryl hemisuccinate in 20 mM HEPES-NaOH, 150 mM NaCl, pH 7.4 supplemented with protease inhibitors and ribonuclease for 2 h at 4 °C. The detergent-solubilized mixture was then centrifuged at 18,000 r.p.m. (39,191 g) for 40 min to pellet any insoluble material. The resulting supernatant was collected and either directly run on sodium dodecyl sulfate–polyacrylamide gel electrophoresis (SDS-PAGE), or the supernatant was fractionated by size-exclusion chromatography on a Superose 6 column to isolate the fractions corresponding to the elution volume of NCLX, which were then run on SDS-PAGE. The gel was visualized with AcquaStain (Bulldog Bio, AS001000) and the gel fractions in the expected molecular mass range of NCLX were excised for MS analysis. The excised gel pieces were washed with H_2_O and 100% ACN, reduced using 50 mM ammonium bicarbonate pH 8.5 (ABC) with 6.5 mM dithiothreitol for 60 min at room temperature, alkylated using 50 mM ABC with 54 mM iodoacetamide for 30 min in the dark at room temperature, and washed with alternating 100% ACN and ABC solutions. To digest the peptides, the dehydrated gel pieces were pre-incubated with digestion buffer (3 ng µl^–1^ trypsin in ABC) for 60 min on ice. The excess digestion buffer was then replaced with an equivalent volume of ABC, and the samples were incubated overnight at 37 °C for protein digestion. The gel pieces were treated with 100% ACN to extract the peptides, which were dried in a vacuum concentrator and resuspended in 10 μl of 0.2% FA, 3% ACN for LC–MS/MS analysis with parallel reaction monitoring (PRM).

### LC–MS/MS analysis

Data-dependent acquisition (DDA) analysis of purified, tryptic, human NCLX peptides was performed to build a spectral peptide library and to generate the PRM inclusion list yielding expected *m*/*z* and retention time windows for twelve unique NCLX peptides, which was subsequently used to examine the presence of NCLX in WT and KO HeLa samples. 2 µl of reconstituted peptides (~0.57 µg) were loaded onto a Dionex Ultimate 3000 RPLC nano system (Thermo Fisher Scientific) for liquid chromatography using mobile phases A (0.2% FA in water) and B (0.2% FA in ACN). The samples were first processed on an Acclaim PepMap 100 C18 trap column (Thermo Fisher Scientific, 164213) using 100% A at 5 µl min^–1^ flow rate. The peptides were then separated using a nanoflow ultra-high-performance liquid chromatography column (Aurora Ultimate 25 cm, IonOpticks, AUR3-25075C18) with an elution gradient of 2–28% over 66.5 min at 300 nl min^–1^. An Orbitrap Fusion Tribrid MS system (Thermo Fisher Scientific) was used to analyse eluted peptides. A Nanospray Flex Ion Source (Thermo Fisher Scientific) held at +2.2 kV was used to ionize precursors, with an inlet capillary temperature of 275 °C. The MS1 scans for both DDA and PRM methods were collected over 350–1,350 *m*/*z* (resolution = 60,000 at 200 *m*/*z*; automatic gain control (AGC) target = 400,000; RF lens = 30%; maximum injection time = 118 ms; normalized AGC target = 100%). The MS2 scans for both DDA and PRM methods were collected over 140–1,400 *m*/*z* (isolation window = 1.6 *m*/*z*; AGC target = 50,000; maximum injection time = 22 ms; Orbitrap resolution = 15,000 at 200 *m*/*z*; higher-energy collisional dissociation collision energy = 30%). The DDA method used a top 20 precursor pick for MS2 acquisition. The MS2 scans for the PRM method were collected using a targeted mass-triggered scan function using an inclusion list containing reference *m*/*z* and corresponding retention time windows (Supplementary Table 1).

### LC–MS/MS data analysis

Datasets were imported into Skyline (v.23.1.1.520; MacCoss Lab software). The confidence of the peptide identity was assessed by the number of fragment ions, and their retention time compared with the purified NCLX peptides. Confidently identified peptides were determined by the presence of at least three fragment ions (<10 ppm error) within a 4 min retention time window from the purified NCLX peptides. Furthermore, transition ions were compared to library spectrum for the same peptide. Mass spectra for peptides, SLGVVFR and ALNPLDYMK, were obtained using FreeStyle 1.8 SP2 (Thermo Fisher Scientific).

### Cell culture and molecular biology

Chinese hamster ovary, HEK293 and HeLa cells were cultured in DMEM with 10% fetal bovine serum. HCT116 cells were cultured in McCoy’s 5A medium with 10% fetal bovine serum. All of these vertebrate cell lines were incubated at 37 °C with 5% CO_2_. Sf9 cells were cultured in the Sf-900 III SFM medium at 27 °C.

To express human NCLX in HEK cells, a gene encoding a C-terminally 1D4 (TETSQVAPA)-tagged full-length NCLX was cloned into the pcDNA3.1(+) vector. Site-directed mutagenesis was performed using the QuickChange II kit (Agilent) and verified using Sanger sequencing. Transient expression was achieved using Lipofectamine 3000 (Thermo Fisher Scientific) following the manufacturer’s instructions. HEK cells were harvested for experiments two days after transfection. The Bac-to-Bac baculovirus expression system (Thermo Fisher Scientific) was used to express proteins in Sf9 cells. Briefly, C-terminally 1D4-tagged human NCLX or an MCU–EMRE fusion protein^85^ was cloned into the pFastBac1 vector, which was transformed into DH10Bac competent cells to produce bacmids. Sf9 cells were transfected with bacmids using the Cellfectin II reagent to generate the P1 baculovirus, which was then used to infect Sf9 cells to produce the P2 virus. Sf9 cells were harvested for experiments three days after being infected with the P2 virus.

To express human NCLX in *Xenopus* oocyte plasma membranes, the first 28 amino acids in the protein’s N-terminal mitochondrial targeting sequence were substituted with an MAGRQHGSGRLWALGG sequence from the mouse TMIE protein, which was found to show very high levels of oocyte plasma membrane expression. The DNA sequence was then optimized for *Xenopus* expression and cloned into a pOX vector for in vitro RNA synthesis using the mMESSAGE mMACHINE T3 transcription kit (Thermo Fisher Scientific) as described in our previous work^86^.

CRISPR KO was performed as described in a past work^87^. The sgRNA sequences used for HeLa and CHO cells are 5′-CCTGGATCTACCAACGGCAA-3′ and 5′-GGCTACCTGGACTACCTCGA-3′, respectively. To confirm gene KO, we cloned (1) the NCLX gene and (2) the NCLX complementary DNA produced by reverse transcription of the NCLX mRNA into pcDNA3.1(+) for Sanger sequencing to identify the exact gene modifications. qPCR was also performed in these *NCLX* KO lines, showing reduced NCLX mRNA levels, consistent with nonsense-mediated mRNA decay caused by CRISPR-induced premature stop codons (Extended Data Fig. 8g). Forward and reverse primers for HEK and HeLa cells: GGCTTCACTGGCTC TTTGCTT and CGAGAAGGCATCTCCAATGCTGTT, respectively; forward and reverse primers for CHO cells: GCATCATTTTCAATATCCTGGTGG and TGAGTTGGAAACACTGAAGC, respectively. In our hands, and as also observed in other laboratories^35,88^, there are currently no useful antibodies against native NCLX. We were therefore unable to further verify *NCLX* KO using Western blot. As an alternative approach to validate *NCLX* KO at the protein level, NCLX peptides were detected by LC–MS/MS (Supplementary Fig. 2), appearing only in WT but not *NCLX* KO HeLa cells.

### Mitochondrial extraction, protease digestion and Western blot

Protein expression in HEK or Sf9 cells was analysed via Western blot detection of proteins in mitochondrial lysates. HEK cell mitochondria extraction was performed using differential centrifugation as described before^85^. To obtain Sf9 mitochondria, 2 × 10^6^ of Sf9 cells were spun down, resuspended in 1 ml of an Sf9 mitochondria isolation buffer (SMIB, 200 mM sucrose, 10 mM Tris, 1 mM EGTA, pH 7.5), and then lysed by passing through a 27-gauge needle for 25 times on ice in the presence of a protease inhibitor cocktail (Thermo Fisher Scientific, PIA32955). The lysate was spun down at 600 g for 10 min. The supernatant was then transferred to a new tube, and spun down at 7,000 g for 10 min. Finally, the pellet was resuspended in 500 μl of SMIB, and spun down again at 7,000 g for 10 min to obtain mitochondrial samples. NCLX expression in *Xenopus* oocytes was quantified by Western blot analysis of oocyte plasma membranes, which were isolated using our published protocols^86^.

To conduct protease digestion experiments, mitochondria were extracted from HEK cells expressing NCLX constructs in a 10-cm dish, and were treated with 800 µl of a hypotonic shock buffer (5 mM sucrose, 5 mM HEPES, 1 mM EGTA, pH 7.2 KOH) on ice for 10 min to produce mitoplasts. After adding 200 µl of a high-salt buffer (750 mM KCl, 100 mM HEPES, 2.5 mM EGTA, pH 7.2 KOH), mitoplasts were pelleted at 17,000 g for 10 min. The isolated mitoplasts were resuspended in tris-buffered saline (TBS), with a small portion used for protein quantification using the bicinchoninic acid assay (Thermo Fisher Scientific, 23227). The samples were treated with 3 µg of proteinase K (Sigma, 70663) or TEV protease (produced in house) per 10 µg of mitoplast proteins. Protease digestion was performed at room temperature for 5 min, and was terminated by adding 0.5 mM of phenylmethylsulphonyl fluoride (Sigma, P7626) and 2 µg ml^–1^ of leupeptin (Sigma, LEU-RO) and pepstatin (Sigma, PEPS-RO) at room temperature for 5 min. The samples were then denatured with an SDS loading buffer for subsequent SDS-PAGE.

To perform Western blot, 10 μg of mitochondrial or mitoplast proteins or membranes from 20 oocytes were separated using SDS-PAGE and transferred to low-fluorescence PVDF membranes (LI-COR), which were blocked in the LI-COR Intercept blocking buffer. The membranes were then incubated with primary antibodies diluted in TBST (that is, TBS + 0.075% Tween-20) at 4 °C overnight, followed by 1 h incubation with fluorescent secondary antibodies, diluted in TBST, at room temperature. Western blot signals were acquired using a LI-COR Odyssey CLx imager, and quantified using the ImageStudio software (v.5.0). Antibodies and dilutions: anti-1D4 (homemade, 100 ng ml^–1^); anti-Tim23 (Santa Cruz, sc-514463, 1:1,000); anti-MCU (Cell Signalling, D2Z3B, 1:10,000); anti-actin (Santa Cruz, sc-68979, 1:2,000); anti-COX2 (Abcam, ab110258, 1:10,000); anti-Histone H3 (Millipore, 05-928, 1:10,000); IRDye 680RD goat anti-mouse secondary antibody (LI-COR, 925-68070, 1:15,000); and IRDye 800CW goat anti-rabbit secondary antibody (LI-COR, 926-32211, 1:10,000).

### Mitochondrial and oocyte Ca^2+^ transport assays

Each mitochondrial Ca^2+^ flux experiment was performed using 2 × 10^7^ of HEK, HeLa, CHO or HCT116 cells, or 2.8 × 10^7^ of Sf9 cells. Cells were suspended in 10 ml of a wash buffer (120 mM KCl, 25 mM HEPES, 2 mM K_2_HPO_4_, 1 mM MgCl_2_, 50 μM EGTA, pH 7.2 KOH), pelleted at 1,500 g for 3 min, and then resuspended in 2.2 ml of a recording buffer (120 mM KCl, 25 mM HEPES, 2 mM K_2_HPO_4_, 5 mM succinate, 1 mM MgCl_2_, pH 7.2 KOH); 2 ml of the cell suspension was then transferred into a stirred quartz cuvette in a Hitachi F-7100 spectrophotometer (excitation = 508 nm; excitation-slit = 2.5 nm; emission = 531 nm; emission-slit = 5 nm; sampling rate = 2 Hz), with 250 nM of CG5N (Thermo Fisher Scientific, C3737) used to report extra-mitochondrial [Ca^2+^], and 30 μM of digitonin (Sigma, D141) used to permeabilize cells. Inhibitors used in these assays include Ru360 (synthesized in house) and CGP-37157 (Cayman, 1561110).

For oocyte Ca^2+^ uptake assays, stage V–VI oocytes were injected with 50 ng of NCLX mRNA, and incubated in an ND96 solution (96 mM NaCl, 2 mM KCl, 2 mM CaCl_2_, 0.5 mM MgCl_2_, 5 mM HEPES, pH 7.4 NaOH) for 3 to 4 days. To measure ^45^Ca^2+^ uptake (Fig. 5d–g), oocytes were washed three times in an oocyte recording buffer (ORB, 100 mM NMG, 5 mM HEPES, pH 7.4 HCl), and finally placed in a density of ten oocytes per 400 µl ORB. To begin the assay, 400 µl of the oocyte-containing solution was mixed well with 100 µl of ORB containing 5 µCi of ^45^Ca^2+^ (Perkin Elmer, NEZ013001MC). The assay was performed at room temperature. At desired time points, the reaction was terminated by transferring ten oocytes into 30 ml of ORB. After washing the oocytes two more times in fresh 30 ml ORB, each oocyte was lysed individually via pipetting and vigorous shaking in 10 ml of a scintillation cocktail for radioactivity measurements using a Beckman LS6500 scintillation counter. To obtain a data point, we first measured ^45^Ca^2+^ in ten individual oocytes, and obtained the median reading. We then excluded oocytes with readings >5-fold higher than the median, which probably reflect sick oocytes with compromised membranes, as well as those with readings <20% of the median, which probably reflect oocytes with failed mRNA injection. The remaining readings, usually from 5–8 oocytes, were averaged and presented as an independent measurement. The results were discarded if there were fewer than five useful readings from ten oocytes.

To test H^+^-coupled ^45^Ca^2+^ transport (Fig. 5h), oocytes one day after mRNA injection were incubated in a counter flux buffer containing 96 mM NaCl, 2 mM KCl, 0.5 mM CaCl_2_, 2 mM MgCl_2_, 2.5 mM HEPES, 5 µCi ml^–1^ ^45^Ca^2+^, pH 7.4 NaOH at a density of ten oocytes per 1 ml. After two days of equilibration, oocytes were transferred into microcentrifuge tubes, with the external solution reduced to 20 oocytes per 800 µl. To begin the reaction, 800 µl of the oocyte-containing solution was mixed well with 200 µl of counter flux buffer that contains high concentrations of pH buffers (200 mM MOPS for pH 6.8, 200 mM HEPES for pH 7.4, or 200 mM Tris for pH 8.2) to adjust the pH, BAPTA to reduce free [Ca^2+^] to 250 nM, and 5.2 µCi ml^–1^ ^45^Ca^2+^. Reaction termination and data analyses were performed as above, but with 20 oocytes used for each data point (experiments with fewer than ten useful oocyte readings were discarded).

### Reporting summary

Further information on research design is available in the Nature Portfolio Reporting Summary linked to this article.

## Online content

Any methods, additional references, Nature Portfolio reporting summaries, source data, extended data, supplementary information, acknowledgements, peer review information; details of author contributions and competing interests; and statements of data and code availability are available at 10.1038/s41586-025-09491-0.

## Supplementary information

Supplementary Information Supplementary Figs. 1 and 2, and Supplementary Tables 1 and 2.

Reporting Summary

## Source data

Source Data Fig. 5 and Source Data Extended Data Figs. 5 and 8

## Figures and Tables

**Extended Data Table 1:**
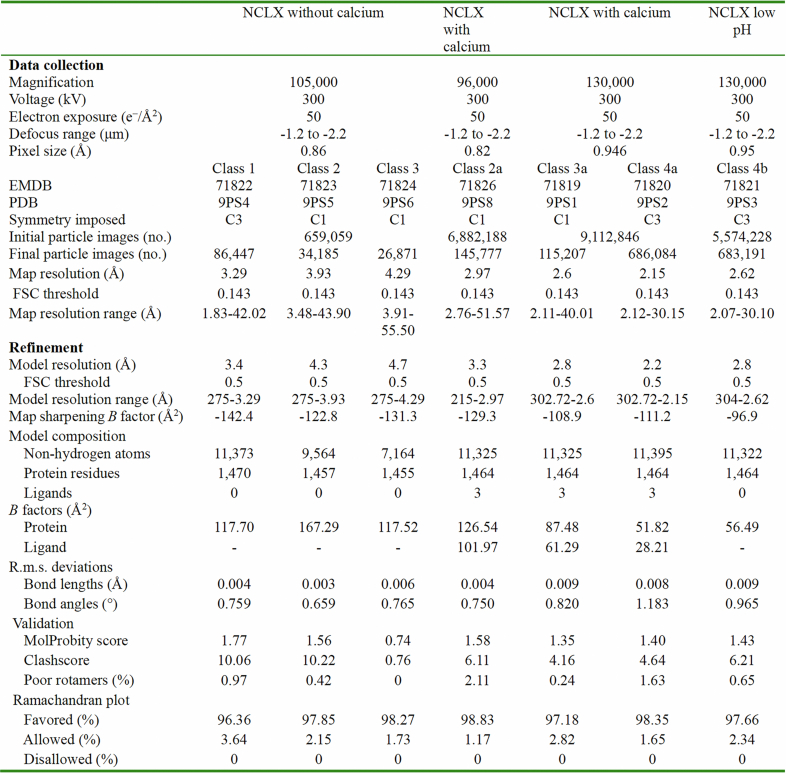
Cryo-EM data collection, refinement, and validation statistics

Cryo-EM data collection, processing, structure determination and validation.

## Data Availability

The NCLX maps have been deposited in the Electron Microscopy Data Bank under codes EMD-71819, EMD-71820, EMD-71821, EMD-71822, EMD-71823, EMD-71824 and EMD-71826. The corresponding models have been deposited in the Protein Data Bank (PDB) under IDs 9PS1, 9PS2, 9PS3, 9PS4, 9PS5, 9PS6 and 9PS8. Mass spectrometry data have been deposited in MassIVE under MSV000098428. Simulation trajectories generated in this study are available at 10.5281/zenodo.15793477 (ref. ^89^). The PDB and OPM files for 3V5U used in this study are available from the PDB (ID: 3V5U) and the OPM database (https://opm.phar.umich.edu/proteins/1933), respectively. Source Data are provided with this paper.
