# Supplementary material for: Structure and mechanism of the mitochondrial calcium transporter NCLX
Source: Nature. 2025 Sep 10;646(8087):1272–80. doi: 10.1038/s41586-025-09491-0 (PMC12571890; doi:10.1038/s41586-025-09491-0)
Supplement: Supplementary file 1 — Supplementary Figs. 1 and 2, and Supplementary Tables 1 and 2. [file 41586_2025_9491_MOESM1_ESM.pdf]

nature portfolio

https://doi.org/10.1038/s41586-025-09491-0

Supplementary information

Structure and mechanism of the mitochondrial calcium transporter NCLX

In the format provided by the authors and unedited

Supplementary Figure 1 | Source images for gel

Gel source data for Figure 5b

These two images originate from the same scan but were pseudocolored in red and green to highlight NCLX and Tim23, respectively.

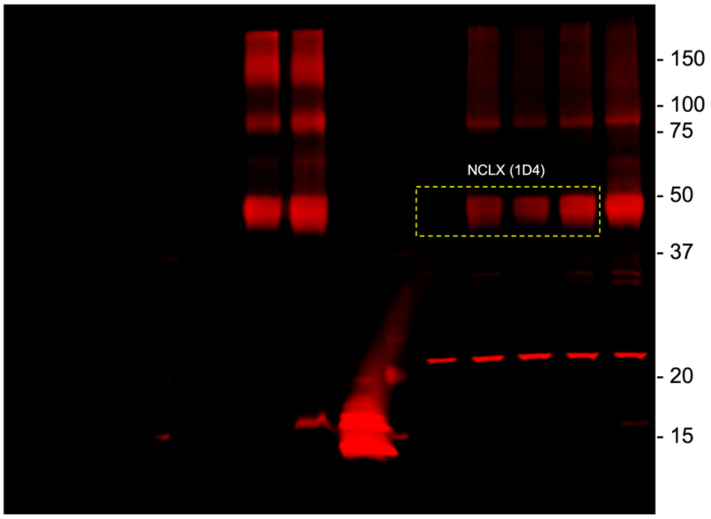

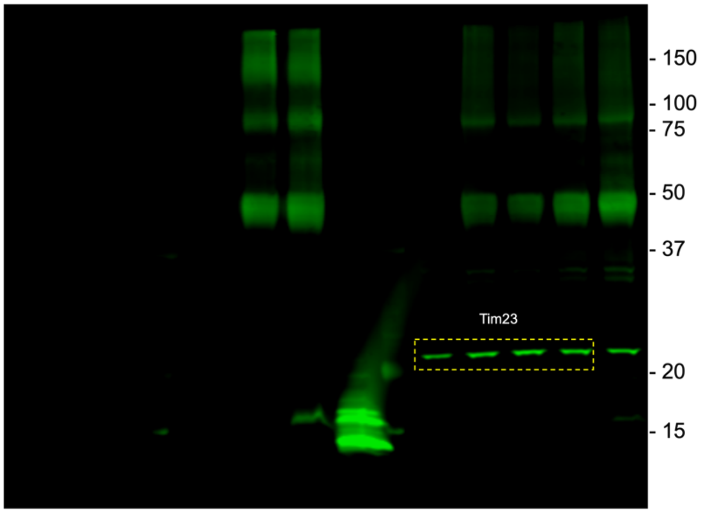

Gel source data for Figure 5c

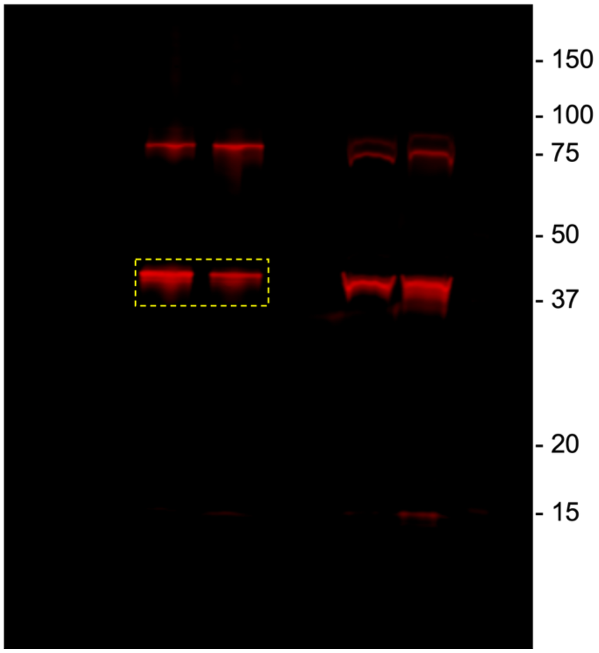

Gel source data for Extended Data Figure 2b

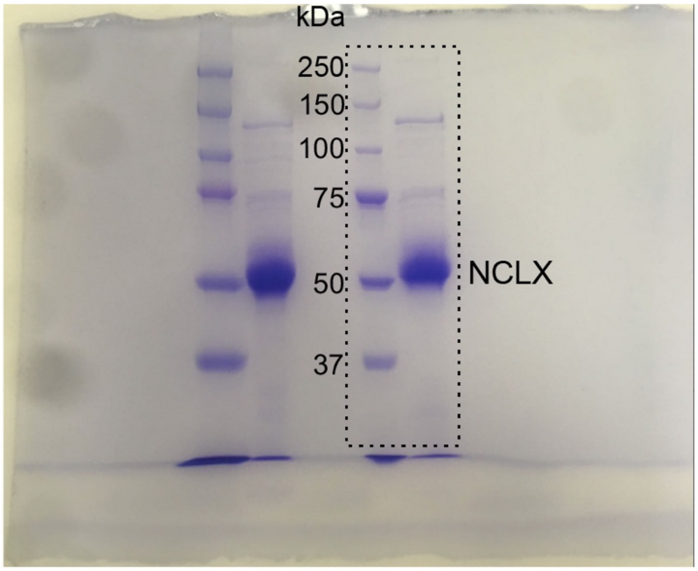

Gel source data for Extended Data Figure 5a

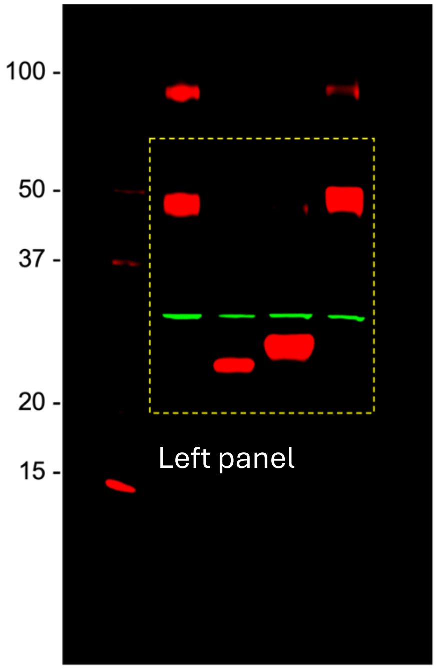

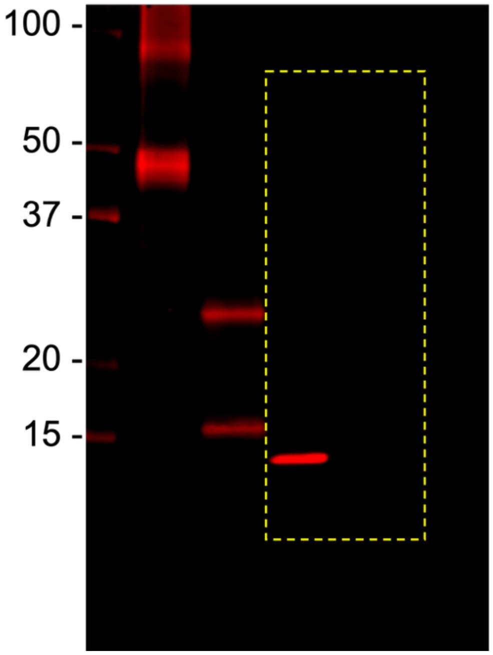

Gel source data for Extended Data Figure 5c:

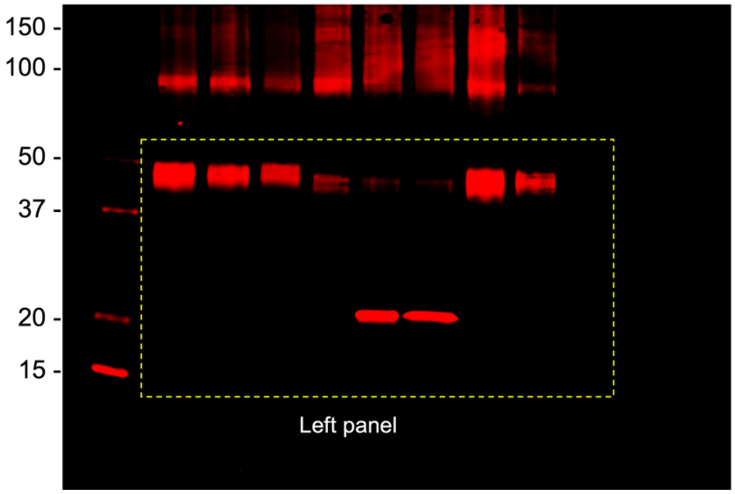

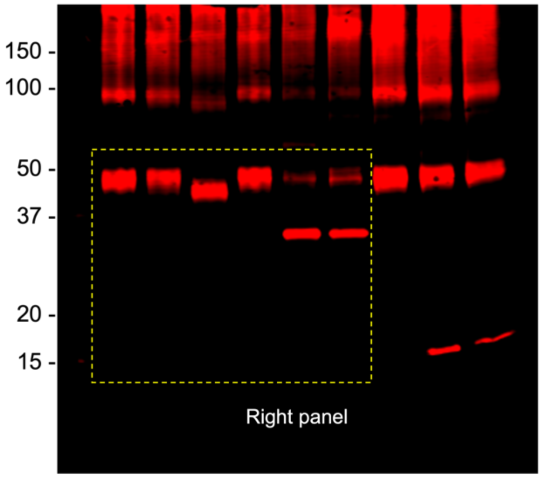

Gel source data for Extended Data Figure 8a:

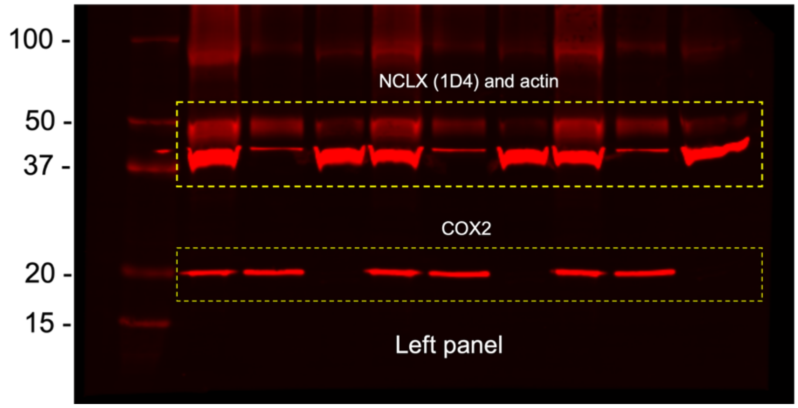

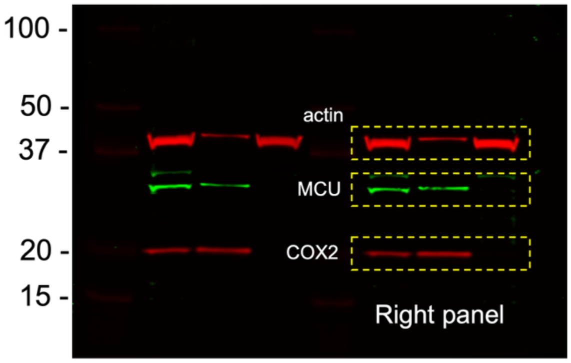

Gel source data for Extended Data Figure 8b:

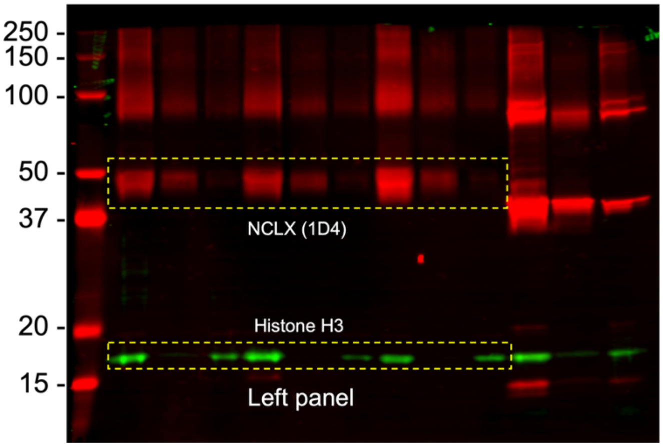

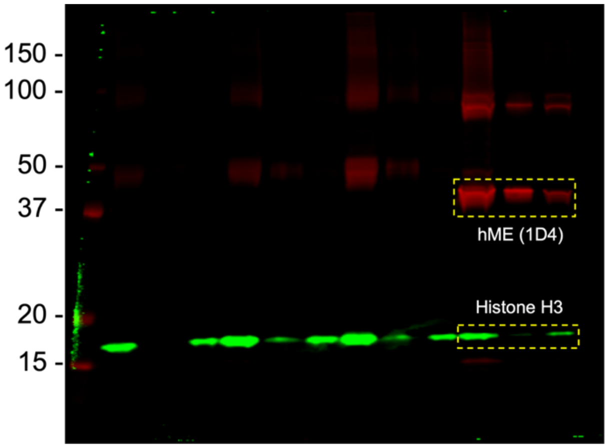

Gel source data for Extended Data Figure 8d:

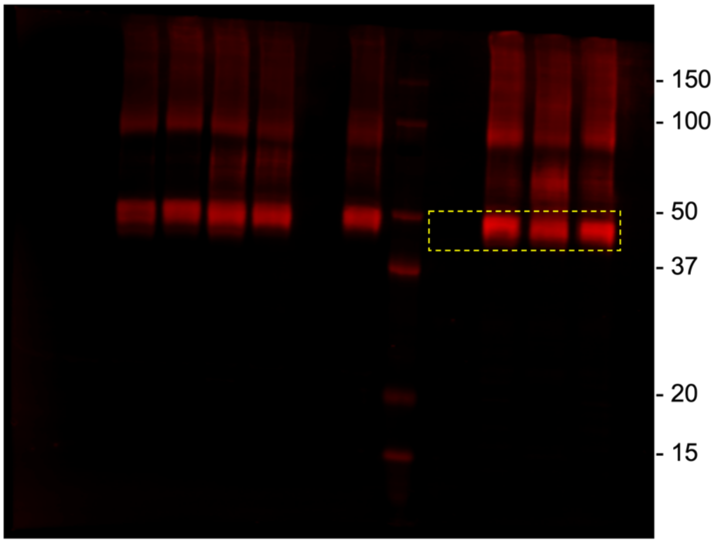

Gel source data for Extended Data Figure 8e:

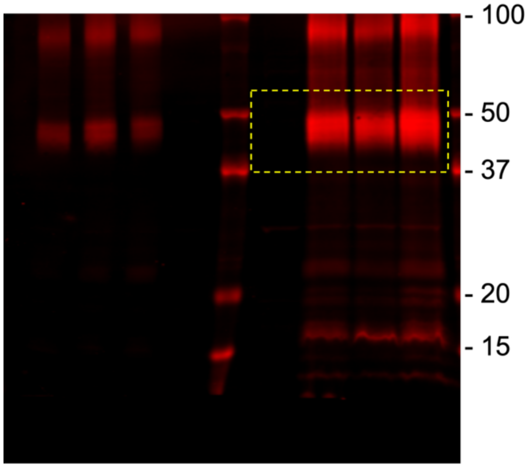

a

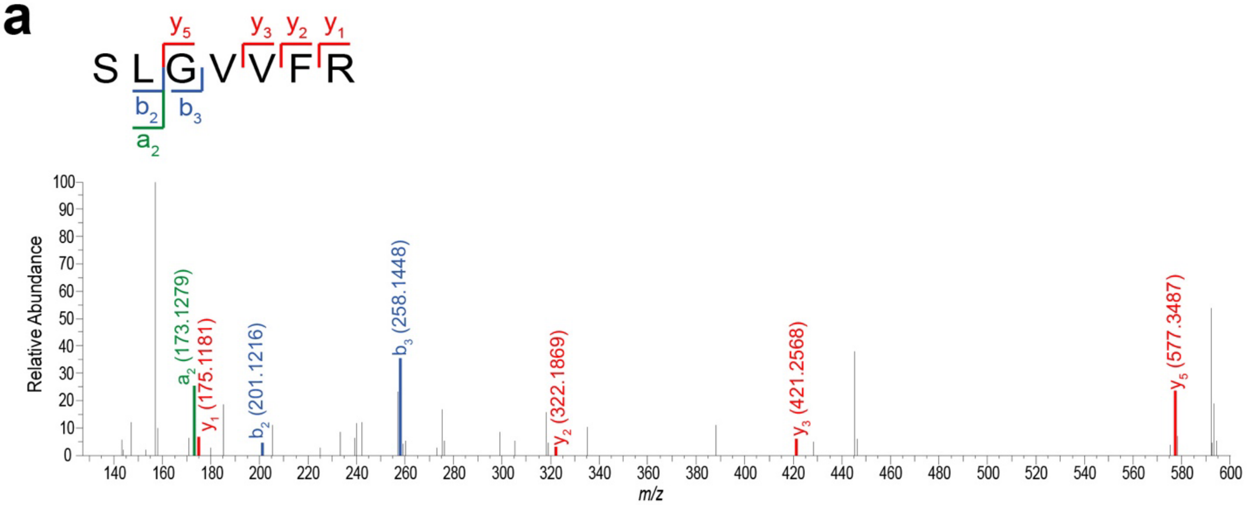

b

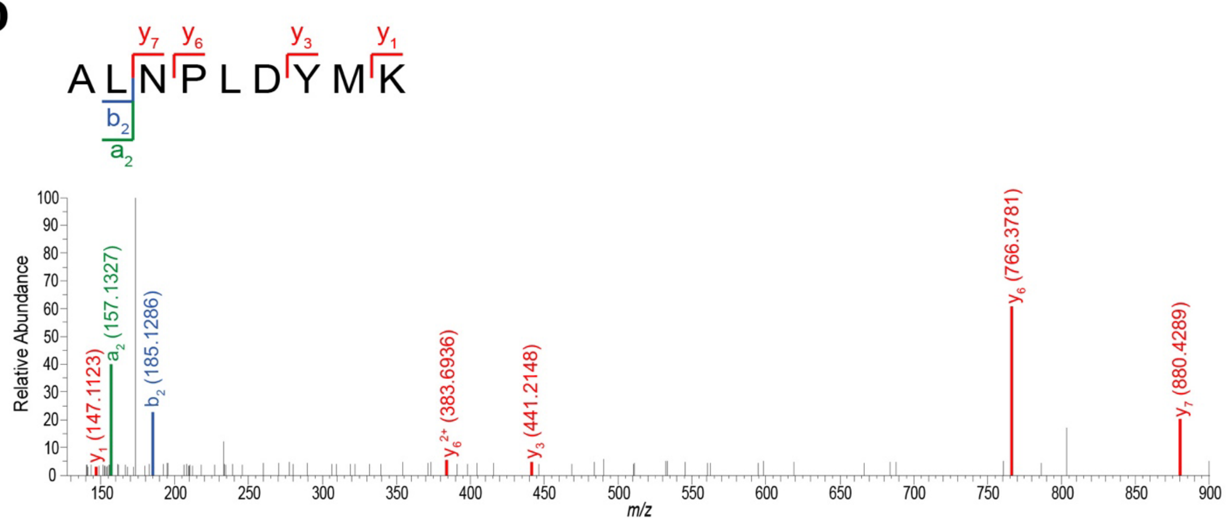

Supplementary Figure 2 | Tandem mass spectra of NCLX peptides from LC-MS/MS PRM analysis of fractionated mitochondrial proteins from WT HeLa cells. Extracted mass spectra of peptides (**A**) SLGVVFR (*m/z* 389.2345, *z* = 2+) and (**B**) ALNPLDYMK (*m/z* 532.7759, *z* = 2+) from WT cells are presented. Observed spectra for a, b, and y ions are indicated in green, red, and blue, respectively. NCLX peptides were detected in WT cells in two independent experiments: the first used total solubilized mitochondria fractionated on SDS-PAGE (shown above; both peptides A and B were detected), and the second used solubilized mitochondria fractionated on size-exclusion chromatography prior to SDS-PAGE fractionation (peptide A was detected). Neither peptide was detected in NCLX-KO cells. Mass spectra from KO cells are not shown due to absence of peptide identifications. No additional NCLX peptides were identified in either WT or KO cells.

Peptide	m/z	z	RT Time (min)	Window (min)
K.DDQNWK.R	403.1774	2	19.76	5
K.FFC[+57.02146]PNLSAISTTLK.L	799.916	2	66.35	5
K.LPVEFLLLLTVPVVDPDK.D	669.7307	3	75.93	5
R.ALNPLDYM[+15.99492]K.W	540.7733	2	42.3	20
R.ALNPLDYMK.W	532.7759	2	55.5	20
R.ALNPLDYMKWR.R	469.5798	3	62.37	20
R.ALNPLDYMKWR.R	703.8661	2	62.39	20
R.ALNPLDYMKWRR.K	521.6135	3	55.77	20
R.DIVFYMVAVFLTFLMLFR.G	742.4016	3	77.99	5
R.GSLFC[+57.02146]PMPVTPEILSDSEEDR.V	1190.046	2	69.94	20
R.GSLFC[+57.02146]PMPVTPEILSDSEEDR.V	793.6996	3	70	20
R.SLGVVFR.L	389.2345	2	45.88	20
R.VSSNTNSYDYGDEYR.P	885.3661	2	31.99	20
R.VSSNTNSYDYGDEYRPLFFYQETTAQILVR.A	1192.902	3	73	20
K.LPVEFLLLLTVPVVDPDK.D	1004.093	2	76	20
R.LSNTVLGLTLLAWGNSIGDAFSDFTLAR.Q	984.8537	3	76.2	20
R.GSLFCPMPVTPEILSDSEEDR.V	1198.043	2	75.7	20

Supplementary Table 1 | Parallel reaction monitoring parameters for NCLX peptides

Supplementary Table 2 | Simulations Setup Details

Box Dimensions*	120 Å x140 Å x110 Å
Salt Concentration	75 mM calcium ions, 150 mM chloride ions
Total Atoms*	135,000
Total Waters*	29,000
Total Lipids*	300
Lipid Type	palmitoyl-oleoyl-phosphatidylcholine (POPC)

*Approximate values are listed for box dimensions, total atoms, total waters, and total lipids, as precise values vary between simulation conditions.
