# Supplementary material for: Structure and mechanism of the mitochondrial calcium transporter NCLX
Source: Nature. 2025 Sep 10;646(8087):1272–80. doi: 10.1038/s41586-025-09491-0 (PMC12571890; doi:10.1038/s41586-025-09491-0)
Supplement: Supplementary file 2 — Reporting Summary [file 41586_2025_9491_MOESM2_ESM.pdf]

nature portfolio

Corresponding author(s): Chen-Wei Tsai, Ming-Feng Tsai, and Liang Feng

Last updated by author(s): Jul 29, 2025

Reporting Summary

Nature Portfolio wishes to improve the reproducibility of the work that we publish. This form provides structure for consistency and transparency in reporting. For further information on Nature Portfolio policies, see our Editorial Policies and the Editorial Policy Checklist.

Statistics

For all statistical analyses, confirm that the following items are present in the figure legend, table legend, main text, or Methods section.

n/a	Confirmed	
☐	☒	The exact sample size (*n*) for each experimental group/condition, given as a discrete number and unit of measurement
☐	☒	A statement on whether measurements were taken from distinct samples or whether the same sample was measured repeatedly
☐	☒	The statistical test(s) used AND whether they are one- or two-sided<br>*Only common tests should be described solely by name; describe more complex techniques in the Methods section.*
☒	☐	A description of all covariates tested
☒	☐	A description of any assumptions or corrections, such as tests of normality and adjustment for multiple comparisons
☐	☒	A full description of the statistical parameters including central tendency (e.g. means) or other basic estimates (e.g. regression coefficient) AND variation (e.g. standard deviation) or associated estimates of uncertainty (e.g. confidence intervals)
☐	☒	For null hypothesis testing, the test statistic (e.g. *F*, *t*, *r*) with confidence intervals, effect sizes, degrees of freedom and *P* value noted<br>*Give P values as exact values whenever suitable.*
☒	☐	For Bayesian analysis, information on the choice of priors and Markov chain Monte Carlo settings
☒	☐	For hierarchical and complex designs, identification of the appropriate level for tests and full reporting of outcomes
☒	☐	Estimates of effect sizes (e.g. Cohen's *d*, Pearson's *r*), indicating how they were calculated

Our web collection on statistics for biologists contains articles on many of the points above.

Software and code

Policy information about availability of computer code

Data collection	SerialEM 3.7, EPU 3.2
Data analysis	Phenix 1.19, MolProbity 4.4, CryoSPARC 4.2, CryoSPARC 4.4, Chimera 1.17.3, Coot 0.9.8, MotionCor2 1.2.1, ChimeraX 1.9, Topaz 0.2.4, DeepEMhancer (https://github.com/rsanchezgarc/deepEMhancer), PyMOL 2.5, VMD 1.9.3, AMBER20, AmberTools17, Prime (implemented in Maestro v 12.8.117), Dabble (v 2.7.9); SHAKE (implemented in AMBER20), Skyline version 23.1.1.520, FreeStyle™ 1.8 SP2, ImageStudio (version 5.0).

For manuscripts utilizing custom algorithms or software that are central to the research but not yet described in published literature, software must be made available to editors and reviewers. We strongly encourage code deposition in a community repository (e.g. GitHub). See the Nature Portfolio guidelines for submitting code & software for further information.

Data

Policy information about availability of data

All manuscripts must include a data availability statement. This statement should provide the following information, where applicable:

- Accession codes, unique identifiers, or web links for publicly available datasets
- A description of any restrictions on data availability
- For clinical datasets or third party data, please ensure that the statement adheres to our policy

The NCLX maps have been deposited in the Electron Microscopy Data Bank under EMD-71819, EMD-71820, EMD-71821, EMD-71822, EMD-71823, EMD-71824 and

EMD-71826. The corresponding models have been deposited in the Protein Data Bank under 9PS1, 9PS2, 9PS3, 9PS4, 9PS5, 9PS6 and 9PS8. Mass spectrometry data have been deposited in MassIVE under MSV000098428. Simulation trajectories generated in this study are available at https://doi.org/10.5281/zenodo.15793477. The PDB and OPM files for 3V5U used in this study are available from the Protein Data Bank (PDB ID: 3V5U) and the OPM database (https://opm.phar.umich.edu/proteins/1933), respectively.

Research involving human participants, their data, or biological material

Policy information about studies with human participants or human data. See also policy information about sex, gender (identity/presentation), and sexual orientation and race, ethnicity and racism.

Reporting on sex and gender	N/A.
Reporting on race, ethnicity, or other socially relevant groupings	N/A.
Population characteristics	N/A.
Recruitment	N/A.
Ethics oversight	N/A.

Note that full information on the approval of the study protocol must also be provided in the manuscript.

Field-specific reporting

Please select the one below that is the best fit for your research. If you are not sure, read the appropriate sections before making your selection.

☒ Life sciences ☐ Behavioural & social sciences ☐ Ecological, evolutionary & environmental sciences

For a reference copy of the document with all sections, see nature.com/documents/nr-reporting-summary-flat.pdf

Life sciences study design

All studies must disclose on these points even when the disclosure is negative.

Sample size	No statistical methods were used to pre-determine sample sizes, but our sample sizes are comparable to those reported in previous publications (PMID: 37036971; 29891485; 27099988). The data size for cryoEM was determined by the availability of the microscope time and the particle density on the grids. Sufficient cryo-EM data were collected to achieve the reported resolution of map, which is sufficient for model building.
Data exclusions	CryoEM data processing involved removing poor-quality or damaged particles to achieve high resolution maps through pre-established standard data classification procedures. We excluded certain data points in Xenopus oocytes 45Ca2+ uptake experiments. Specifically, those oocytes with 45Ca2+ readings higher than 5-fold of the median or lower than 20% of the median are excluded, as the former likely reflects oocytes with compromised membranes while the latter likely reflects oocytes with low or no NCLX expression.
Replication	Functional and biochemical experiments were performed with at least three independent biological replicates, all of which yielded consistent results. The exact number of replicates is indicated in the figure legends. Cryo-EM data processing was not repeated but is expected to be reproducible following the same procedure.
Randomization	This is not relevant to our study, because no grouping was needed.
Blinding	Investigators were not blinded to group allocation, because no grouping was needed for this study.

Reporting for specific materials, systems and methods

We require information from authors about some types of materials, experimental systems and methods used in many studies. Here, indicate whether each material, system or method listed is relevant to your study. If you are not sure if a list item applies to your research, read the appropriate section before selecting a response.

Materials & experimental systems

n/a	Involved in the study	
☐	☒	Antibodies
☐	☒	Eukaryotic cell lines
☒	☐	Palaeontology and archaeology
☒	☐	Animals and other organisms
☒	☐	Clinical data
☒	☐	Dual use research of concern
☒	☐	Plants

Methods

n/a	Involved in the study	
☒	☐	ChIP-seq
☒	☐	Flow cytometry
☒	☐	MRI-based neuroimaging

Antibodies

Antibodies used

Anti-1D4 (home-made, 100 ng/mL)
Anti-Tim23 (Santa Cruz, sc-514463, Lot #G2222, 1:10,0000)
Anti-MCU (Cell Signaling, 14997, Lot #1, 1:10,000)
anti-actin (Santa Cruz, sc-69879, Lot #C2824, 1:2,000)
Anti-COX2 (Abcam, ab110258, Lot #2101058395, 1:10,000)
Anti-Histone H3 (Millipore, 05-928, Lot #2477921, 1:10,000)
IRDye 680RD goat anti-mouse secondary antibody (LI-COR, 925-68070, Lot #D30418-05, 1:15,000)
IRDye 800CW goat anti-rabbit secondary antibody (LI-COR, 926-32211, Lot #D11215-03, 1:10,000)

Validation

The anti-1D4 antibody was validated in house, using lysates from cells with or without expression of 1D4-tagged proteins, as described previously (DOI: 10.7554/eLife.15545), demonstrating specific detection of a single band corresponding to the 1D4-tagged protein.

The anti-Tim23 antibody (Santa Cruz) was validated by the manufacturer, demonstrating detection of a single ~23 kDa band in Western blot analyses of mouse, rat, and human cell lysates, such as those from MCF7 cells.

The anti-MCU antibody (Cell Signaling) was validated in-house using lysates from wild-type and MCU-knockout HEK293 cells, showing a single band at ~30–35 kDa in wild-type cells that is absent in knockout cells.

The anti-actin antibody (Santa Cruz) was validated by the manufacturer, demonstrating a single 43-kDa band in Western blot analyses of mouse, rat, and human cell lysates.

The anti-COX2 antibody (Abcam) was validated by the manufacturer, showing a single ~25-kDa band in Western blot analysis of human heart tissue and specific staining in immunocytochemistry of human MRC5 cells.

The anti-Histone H3 antibody (Millipore) was validated by the manufacturer, showing a single ~20 kDa band in Western blot analyses of nuclear extracts from HeLa cells or purified recombinant Histone H3.

Eukaryotic cell lines

Policy information about cell lines and Sex and Gender in Research

Cell line source(s)

HEK 293 (ATCC, #CRL-11268); HEK293S (ATCC, #CRL-3022); HeLa (ATCC, #CCL-2); CHO (ATCC, #CCL-61); HCT116 (from Dr. Mohamed Treback, eLife e59686, original source: ATCC, #CCL-247); Sf9 (ATCC, #CRL-1711).

Authentication

Sf9 cells were initially authenticated with short tandem repeat profiling when purchased from ATCC, but were not further authenticated later as these cells grew in different incubators as other mammalian cell lines. HEK293S were authenticated by ATCC using short tandem repeat profiling, and no further authentications were performed for this study as these cells were used for protein overexpression and purification. All other cell lines were authenticated by ATCC using short tandem repeat profiling on an annual basis.

Mycoplasma contamination

All cell lines used for functional studies were checked for mycoplasma contamination semi-annually using PCR with no contamination detected.

Commonly misidentified lines
(See ICLAC register)

None of the cell lines used are listed in the ICLAC database.
